# Molecular identification of major green tide-forming *Ulva* species and their spatiotemporal patterns on the Korean coast

**DOI:** 10.1038/s41598-026-50151-8

**Published:** 2026-05-05

**Authors:** Hye Jin Park, Seo Yeon Byeon, Sang Rul Park, Young Baek Son, Ji Hyoun Kang, Hyuk Je Lee

**Affiliations:** 1 Molecular Ecology and Evolution Laboratory, Department of Biological Science, Sangji University, Wonju, 26339 Korea; 2 Oceanic Climate and Ecology Research Division, National Institute of Fisheries Science, Busan, 46083 Republic of Korea; 3 Estuarine and Coastal Ecology Laboratory, Department of Marine Life Sciences, Jeju National University, Jeju, 63243 Korea; 4 Jeju Marine Research Section, Korea Institute of Ocean Science and Technology, Jeju, 63349 Korea; 5 Korean Entomological Institute, Korea University, Seoul, 02841 Korea

**Keywords:** Climate change, DNA barcoding, Jeju Island, Nutrient input, Seaweed tide, *Ulva* species, Ecology, Ecology, Environmental sciences, Ocean sciences

## Abstract

Green tides – massive proliferations of green macroalgae (*Ulva* spp.) – have increasingly occurred worldwide in recent years, driven by accelerating climate change and anthropogenic nutrient inputs. These blooms disrupt coastal ecosystems, leading to biodiversity loss and economic damage. In Korea, green tides have persisted on Jeju Island since the 2000s, and have also been sporadically reported on the southern mainland coasts. However, the specific *Ulva* species responsible for these blooms and their spatiotemporal dynamics remain largely unknown. Here, we investigated *Ulva* community structure and relative frequencies from 46 sites (966 specimens) on Jeju Island and the southern coasts, using chloroplast *tuf*A gene-based phylogenetic analysis, complemented by additional nuclear 5S rDNA marker. We found considerable differences in *Ulva* community composition between Jeju Island and the southern coasts, along with pronounced seasonal variation. A total of 11 *Ulva* species were identified from both regions, with *Ulva ohnoi* and *U. australis* being dominant on Jeju Island and *U. australis* and *U. linza* prevailed on the southern coasts. Our results provide essential genetic insights into major bloom-forming *Ulva* species and their spatiotemporal dynamics in order to support effective management of green tide events in Korean coastal ecosystems.

**Supplementary Information:**

The online version contains supplementary material available at 10.1038/s41598-026-50151-8.

## Introduction

Due to the recent acceleration of climate change, macroalgal blooms have been occurring more frequently in many coastal regions worldwide^1–4^. Since the 1990s, large-scale coastal macroalgal outbreaks have become almost routine events, accompanied by a rapid rise in global concerns^2,4^. More than two hundred-sixty publications have been documented for bloom-forming macroalgal species worldwide to date^5^. Among these recurrent events, red algal bloom (e.g. Rhodophyta *Trichogloeopsis pedicellata*), green tide, and golden tide represent the most commonly reported incidences of macroalgal blooms^5^. A macroalgal bloom caused by the red alga *T. pedicellata* was reported in the southwestern Caribbean Sea after a severe hurricane event^6^. The terms, green tide and golden tide, collectively referred to as seaweed tide were coined to describe seawater that appears as a ‘green’ or ‘brown’ carpet (or paint) due to masses of floating seaweeds. These phenomena are primarily caused by green macroalgae (Chlorophyta) and brown macroalgae (Phaeophyceae), both of which have high growth and proliferation rates associated with their unique life history traits^7^. Consequently, green and golden tides have become critical ecological and environmental issues worldwide^8^. Although multiple ecological and environmental drivers have been proposed, the mechanisms and relative importance of these factors underlying large-scale bloom formation remain still poorly understood^9^. Green tides are predominantly formed by the genus *Ulva*, including formerly classified the genus *Enteromorpha*^10^, whereas golden tides are caused by *Sargassum* species. In the northwestern Pacific, golden tides are known to be exclusively involved with *Sargassum horneri*^11^.

Coastal areas such as estuaries, harbors and bays, where seawater is partially enclosed by natural or artificial barriers, are more prone to green macroalgal blooms (i.e. green tides). The rise in green macroalgal biomass in these regions is largely attributed to human-driven factors, such as nutrient enrichment, eutrophication and pollution^9^. Large-scale green tide events caused by rapid proliferation of *Ulva* species can severely impact coastal ecosystem and local economy^2–4,9^. Previous studies have suggested that human-driven processes, such as aquaculture, coastal development, and wastewater discharge, increase inorganic nutrient inputs (e.g. dissolved inorganic nitrogen and phosphorus), leading to coastal eutrophication and ultimately promoting green tide formation^[,12^. However, although nutrient loading, particularly nitrate enrichment has often been identified as an important driver, increases in algal biomass are not always directly coupled to ambient nutrient concentrations. In some regions, the spatial extent of green tides has continued to expand even as nitrogen and phosphorus concentrations have decreased, suggesting that species-specific growth and reproductive traits of dominant *Ulva* taxa also play a key role^13^. Understanding the possible causes and environmental factors underlying green tide formation is essential for developing mitigation and management strategies to reduce their negative impacts^1,2,4^.

The genus *Ulva* is divided into two major morphological types – foliose and tubular. The tubular form was previously classified as the genus *Enteromorpha* but was later synonymized with *Ulva* based on genetic evidence^14^. *Ulva* species exhibit two distinct growth forms, such as attached or free-floating thalli^15^. Because of their high level of phenotypic plasticity in morphology, which varies with habitat environmental conditions, accurate species identification based solely on morphology is extremely difficult^16^. For example, among species inhabiting the southern coasts of Korea and Jeju Island (located off the southernmost region of the Korean Peninsula), *Ulva australis* (synonym; *Ulva pertusa*) and *Ulva ohnoi* exhibit similar thallus morphologies, making them difficult or impossible to distinguish morphologically, requiring molecular methods for reliable species identification^17^. Therefore, discrepancies likely exist between reported and actual species numbers, and *Ulva* species diversity may be underestimated due to phenotypic plasticity and incomplete molecular dataset^18,19^. For *Ulva* species with simple thallus structures and lacking differentiated reproductive organs, morphological species identification is particularly challenging^[,20^.

Molecular marker-based studies on green tides are necessary for identifying the major *Ulva* species responsible for these large-scale outbreaks. In the northwestern Pacific, encompassing the coastal regions of China, Japan, and Korea, molecular analyses have been widely applied to investigate the green tide phenomena. The chloroplast-encoded elongation factor Tu (*tuf*A) and the internal transcribed spacer (ITS) region of nuclear ribosomal DNA (nuDNA) are among the most commonly used genetic markers for studying *Ulva* diversity^18,21^. These markers have become popular and have significantly contributed to our understanding of genetic diversity and phylogenetic relationships among *Ulva* species. Moreover, the nuclear 5S ribosomal DNA (5S rDNA) marker has shown great potential for inferring interspecies phylogeny, particularly within the *Ulva linza*-*procera*-*prolifera* (LPP) complex or LPP clade^22–26^. The chloroplast-encoded ribulose-1,5-bisphosphate carboxylase large subunit gene (*rbc*L) has also been employed for DNA barcoding of *Ulva* species^21^. Among these markers, *tuf*A has been widely used since 2010 because it provides higher taxonomic resolution than *rbc*L and exhibits greater amplification success than ITS^27^. However, species delimitation within the LPP clade remains challenging, particularly when using commonly applied molecular markers such as *tuf*A. Therefore, in this study, we combined *tuf*A and 5S rDNA analyses to identify *Ulva* species^25,26^.

In Korean waters, local green tide has been observed year-round, particularly along the northeastern coast of Jeju Island since the 2000s^28^^[,29^. More recently, green tides have also occurred sporadically along the southern coasts^30^. Nevertheless, the *Ulva* species primarily responsible for these blooms remain largely unidentified (but see^18,31)^. Furthermore, potential geographic and seasonal variation in *Ulva* community structure, species diversity and distributional patterns remain poorly understood.

In the present study, we aimed to identify *Ulva* species primarily responsible for green macroalgal blooms along the Korean coast. Specifically, we assessed *Ulva* community structure, species composition and diversity, and seasonal variation on Jeju Island and the southern coasts based on molecular phylogenetic analyses. By applying combined analysis of *tuf*A and 5S rDNA, we determined the dominant *Ulva* species associated with green tides. The objectives of this study were to (1) identify the primary *Ulva* species contributing to local green tide events on Jeju Island and the southern coasts of Korea; (2) investigate geographic variation in *Ulva* community structure, species composition and diversity between the two regions, with a focus on determining whether distinct *Ulva* assemblages underlie regional differences in green tide occurrences; (3) identify temporal (seasonal) variation in *Ulva* community structure to determine whether dominant bloom-forming species exhibit distinct seasonal dominance patterns. Bloom-forming species are considered *Ulva* species that repeatedly dominate green tide events and contribute substantially to macroalgal bloom formation. This study provides a comprehensive understanding of the major *Ulva* species driving green tides along the Korean coast and demonstrates how *Ulva* species composition changes across spatial and temporal scales. The findings of this study lay the foundation for a scientific framework to support the long-term monitoring and management of green tides along the Korean coast.

## Methods

### Sample collection and pretreatment

**Fig. 1:**
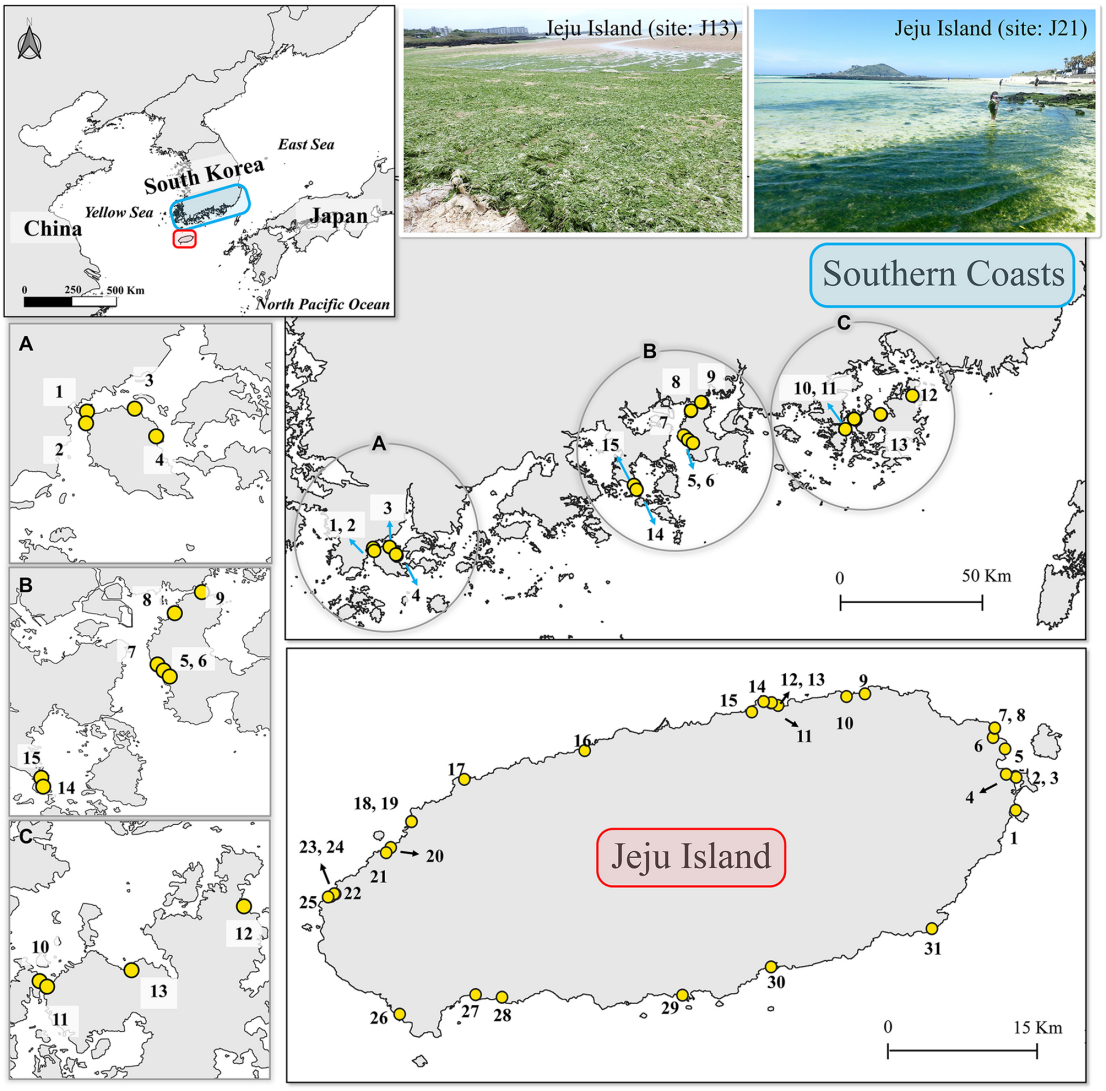
Map showing collection sites for *Ulva* species along the coast of Jeju Island and the southern coasts of Korea. Thirty-one and 15 sampling locations on Jeju Island and the southern coasts, respectively are indicated. *Ulva* specimens were collected on a seasonal basis and thus they were sampled for every site four times.

**Fig. 2:**
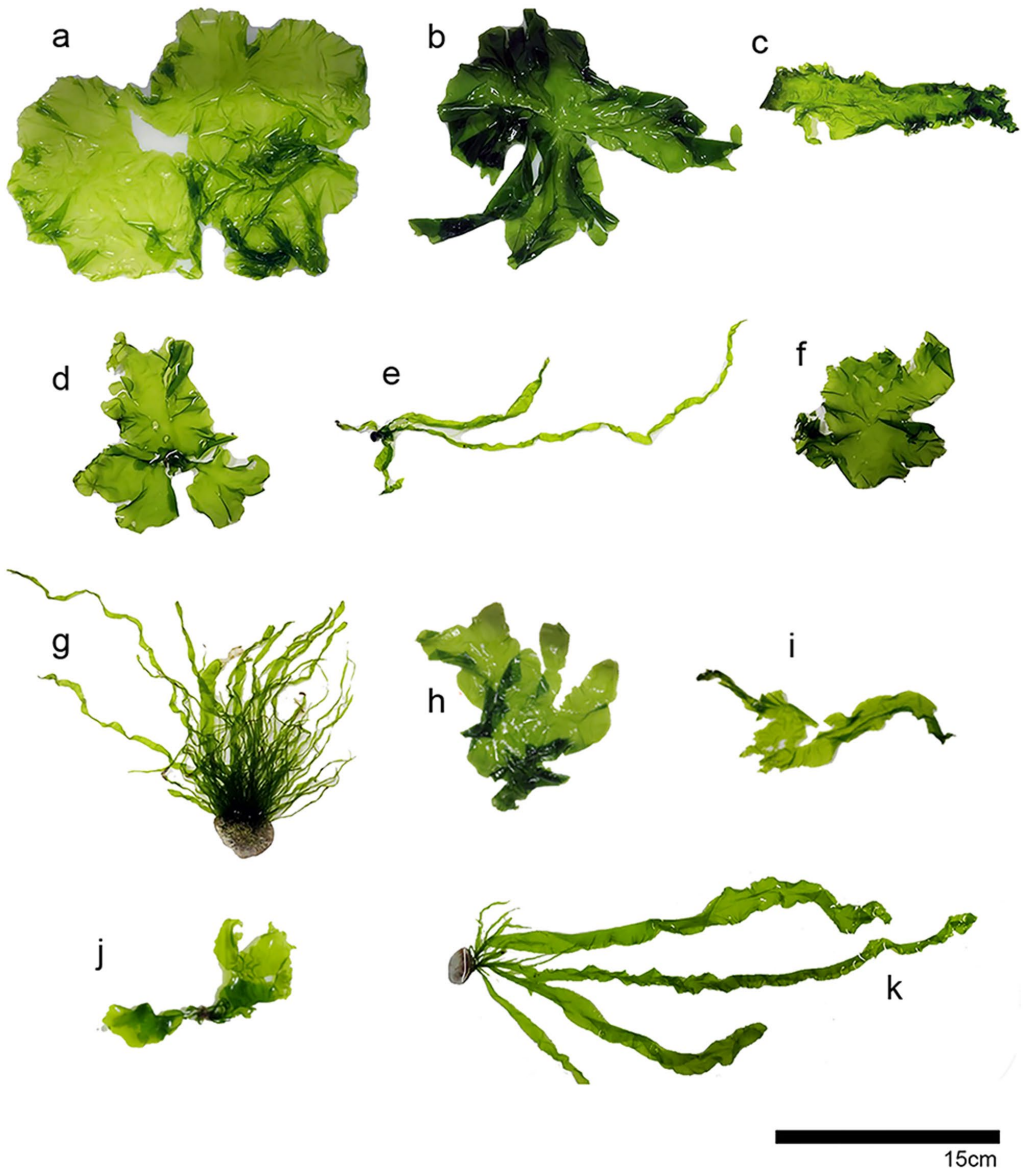
Representative morphological features of *Ulva* species identified based on *tuf*A phylogenetic analysis (^16^; this study). The scale bar is 15 cm [**a**. *U. ohnoi*; **b**. *U. arasakii*; **c**. *U. californica*; **d**. *U. australis* (= *U. pertusa*); **e**. LPP clade species (assigned as *U. procera*); **f**. *U. lacinulata*; **g**. *U. aragoënsis*; **h**. *U. lactuca*; **i**. *U. compressa*; **j**–**k**. LPP clade species (*U. prolifera*).].

This study was conducted on a total of 966 specimens at 46 locations along the southern coastal areas of the Korean Peninsula (the southern coasts hereafter) and the coastline of Jeju Island, which is located ~ 150–200 km off the southern coast of the mainland, from November 2019 to February 2021 (Fig. 1; Tables S1, S2). Six hundred-twelve specimens from 31 sites and three hundred fifty-four specimens from 15 sites were used for Jeju Island and the southern coasts, respectively for genetic analysis. These sites were selected as the study sites because local green tides have been frequently observed there. Samples were collected on a seasonal basis (spring, summer, autumn, and winter), and information on latitude/longitude for the sampling sites is given in Tables S1, S2. Because the sampling periods differed between Jeju Island (November 2019–February 2021) and the southern coasts (January–October 2021), direct month-to-month comparisons between regions were not conducted. Instead, seasonal patterns or dynamics were analyzed separately within each region, and regional comparisons focused on overall community composition and seasonal patterns of dominant species rather than synchronous temporal dynamics. More than five individuals were collected per morphotype of *Ulva* spp. at each of the sampling sites (Fig. 2) and all specimens were collected at 2–3 m intervals of each other within sites. The sampling distances were kept across sites in Jeju Island and the southern coasts. These sampling schemes would allow to avoid the inadvertent collection of the same individuals^18^. In cases where the sampling areas were geographically separated by a coastal embankment, the inland site was designated as ‘in’ and the coastal site as ‘outside’.

Environmental data [including sea surface temperature (SST), salinity, dissolved inorganic nitrogen (DIN), and etc.] were only obtained from Ministry of Environment Information System (MEIS), Ministry of Oceans and Fisheries in Korea for a period of February 2021 (Fig. S1). These information was, however, only used to provide regional environmental context rather than to infer direct causal relationships with *Ulva* bloom formation.

The collected samples were transported to the laboratory in an insulated cool box containing ice packs to ensure that temperature remained below 4°C during transport. All samples were photographed before DNA extraction. After photographing, the samples were rinsed several times with freshwater to remove any sediment and epiphytic organism and foliose tissue samples were completely dried in a dry oven (Jeio Tech, Korea) at 60°C for at least 24 hours^16,18^. Completely dried samples were powdered using TissueLyserII (Qiagen, USA). The pulverized powder tissue sample was stored with silica gel in a 2.0 ml tube and a Desiccator Cabinet (Kastech, Korea) with an automatic dehumidification function until genetic analysis. Genetic analysis was performed at least for 2–3 individuals per morphotype at each sampling site. A total of 966 specimens were sequenced for the *tuf*A gene. In addition, a subset of 119 individuals belonging to the LPP clade, as identified by *tuf*A, were further analyzed, of which only 105 samples were successfully sequenced for 5S rDNA.

### Genomic DNA extraction, PCR and sequencing

Genomic DNA (gDNA) was extracted using i-genomic Plant DNA Extraction Mini Kit (Intron Biotechnology, Korea) according to manufacturer’s protocol. The concentration of the extracted gDNA was measured using a NanoDrop UV/VIS spectrophotometer (Thermo Fisher Scientific, USA). The cpDNA *tuf*A gene^21^ and nuDNA 5S rDNA were amplified by polymerase chain reaction (PCR). The *tuf*A gene was amplified with the published primers, TufGF4 (5’-GGNGCNGCNCAAATGGAYGG-3’) and TufAR (5’-CCTTCNCGAATMGCRAAWCGC-3’)^,^ and 5S rDNA region was amplified with the primers, 5S-F (5’-GGTTGGGCAGGATTAGTA-3’) and 5 S-R (5’-AGGCTTAAGTTGCGAGTT-3’)^22^. PCR reaction was performed in a total reaction volume of 15 µl with 10×Dream Taq Green buffer (Thermo Fisher Scientific) 1.5 µl, 2.5 mM dNTPs (Bio Basic Inc., Canada) 1.5 µl, 10 pmol forward/reverse primers 0.5 µl, 0.2 units of Taq DNA polymerase (Thermo Fisher Scientific) 0.1 µl, template gDNA (~ 20 ng/µl), and 9.9 µl sterile distilled water using a 2720 thermal cycler (Applied Biosystems, USA). PCR amplification was performed by 35 cycles of initial denaturation at 94 °C for 4 min, denaturation at 94 °C for 1 min, annealing at 45 °C (for *tuf*A) ~ 50 °C (5 S rDNA) for 30–45 s, and extension at 72 °C for 1 min, followed by a final extension reaction at 72 °C for 7 min. The PCR products were visualized by electrophoresis on a 2% agarose gel and purified with enzymatically Exonuclease I and Shrimp Alkaline Phosphatase (New England BioLabs, USA). Sequencing was performed with ABI PRISM 3730xl automated DNA sequencer (Applied Biosystems).

For 5S rDNA region, gel extraction/excision process was undertaken prior to DNA sequencing. The 5S rDNA sequences were used for identifying the species belonging the LPP clade^26^. The resulting DNA fragments were visualized by UV transillumination and analyzed using Gel doc 1000 UV Fluorescent Gel Documentation System-PC (Biorad, USA). After identifying the shortest DNA fragment (about 200–300 bp) in each PCR product of the 5 S rDNA spacer region, PCR yielding band was excised from the gel and purified using MinElute Gel Extraction Kit (Qiagen)^24^.

### Molecular phylogenetic analysis

The 966 *tuf*A DNA sequences obtained were edited using Geneious prime ver. 2021.0.3^32^ and aligned using Clustal Omega ver. 1.2.2^33^. For molecular-based species identification of *Ulva*, 55 haplotypes of 17 species plus two unidentified haplotypes, which were previously determined by *tuf*A phylogenetic analysis^18^, were used as references in this study (Table S3). Two *Blidingia* spp. were used as outgroup (GenBank accession numbers: MK992087, HQ610240). When monophyletic clades were formed in the phylogenetic tree, we defined those as particular “the species”^30^. In contrast, samples that did not cluster with reference-supported clade were designated as *Ulva* sp. (unidentified species). Aligned DNA sequences best fitted the JC (Jukes-Cantor) model based on jModel-test ver. 2.1.7^34^. Neighbor-joining (NJ) analysis was performed using MEGA ver. 7.0^35^ using 1,000 repetitions (bootstrap) with the JC model. Maximum likelihood (ML) analysis was also performed using PhyML ver. 3.1^36^ with 1,000 bootstrap samplings. Based on recent taxonomic revisions supported by type-derived plastid genome and barcode analyses^37^, the lineage previously referred to as *Ulva laetevirens* (often mislabeled as *Ulva rigida*) was reassigned to *Ulva lacinulata*. Accordingly, all *tuf*A haplotypes forming this clade were treated as *U. lacinulata* in the present study, and all synonymous names (*U. laetevirens*, *Ulva armoricana*, and *Ulva scandinavica*) were treated as a single taxon under *U. lacinulata* following current nomenclatural consensus^37^.

To determine species within the LPP clade, additional phylogenetic analysis of the 5S rDNA was conducted on 105 individuals (25 from Jeju Island and 80 from the southern coasts) out of 119 specimens that were assigned to the LPP clade based on the *tuf*A-based phylogenetic analysis. More specifically, the 105 samples identified as *U. procera* or *U. prolifera* (103 *U. procera* and two *U. prolifera*) in *tuf*A-phylogeny were reanalyzed using the 5S rDNA marker with NJ method to distinguish *U. prolifera* and *U. linza*. Previously determined 25 haplotype sequences were used as a reference for this analysis^16^. The total sequence length, including gaps between sequences (24 ~ 125 bp), was 352 bp, and the minimum sequence length of 227 bp.

We further performed model-based algorithmic species delimitation analysis of ABGD (Automatic Barcode Gap Discovery) to corroborate the results of our molecular phylogeny-based species delimitation. ABGD analyses were conducted via the web-interface (https://wwwabi.snv.jussieu.fr/public/abgd/abgdweb.html) with default settings with the exception of the JC model and two relative gap widths (X) (X = 1.0, 1.5) applied^18^. The prior intraspecific diversity (P) was set to range from 0.001 to 0.1. The relative frequency of each *Ulva* species was calculated as the proportion of individuals assigned to each species out of the total number of genetically identified green algal specimens in each region (Jeju Island and the southern coasts). Species identification was based on both *tuf*A and 5S rDNA analyses.

### Community analysis

The species composition ratio of *Ulva* at each site was calculated based on relative abundance data. Prior to analysis, the data were square-root transformed, and Bray–Curtis similarity indices were computed to assess differences in community composition between Jeju Island and the southern coasts. Non-metric multidimensional scaling (NMDS)^38^ was used to visualize spatial patterns in community structure. Statistical significance of spatial differences between the two regions was tested using analysis of similarities (ANOSIM). All analyses and visualizations were conducted using PRIMER-e v7 (PRIMER-E Ltd., Plymouth, UK).

In addition, we tested for significant difference in life-form type (floating vs. benthic [attached] thalli) between the two dominant *Ulva* species (*U. australis* and *U. ohnoi*) in Jeju Island. Differences in the proportions of benthic and floating individuals between the two species (*U. australis* [*N* = 129] and *U. ohnoi* [*N* = 209]) were evaluated using a chi-square test, and a preference index was subsequently calculated following the method Ivlev’s and modified by Jacobs’s^39^.

## Results

### Model-based species delimitation

The number of *Ulva* species groups identified by ABGD based on *tuf*A was consistent with the number of species groups (clusters) inferred from phylogenetic analyses. In the ABGD results, the number of species groups varied depending on the values of P and X. The number of groups showing the minimum difference (i.e. 1.0) between the initial partition (IP) and recursive partition (RP) was 14 (IP) and 15 (RP) when X = 1.0 (*P* = 0.0077), whereas the numbers decreased to 10 (IP) and 11 (RP), respectively when X = 1.5 (*P* = 0.0077). After excluding *Blidingia* spp. and *Ulva* sp. taxa, the number of *Ulva* groups was reduced (Table 1; Table S4).

Under this parameter combination, given the minimum difference between IP and RP (one group), the number of *Ulva* species groups based on *tuf*A was inferred to be ten (when *P* = 0.0077, X = 1.0) (Table 1). The results also showed that increasing X from 1.0 to 1.5 resulted in fewer species groups across all *P* values, indicating that the ABGD results were sensitive to parameter selection. For example, when X = 1.0, 15 groups were detected (*P* = 0.0077), but this number decreased to 11 at X = 1.5 when *P* = 0.0077. Some species groups, such as *U. ohnoi*, *U. californica* and *U. compressa* were consistently identified across different parameter settings, whereas *U. prolifera* and *U. procera* showed variable groupings. In addition, *U. lactuca* and *U. aragoënsis* represented in the phylogenetic analyses (see below) were not recovered as independent partitions in the ABGD results, which is perhaps because their *tuf*A sequences showed low genetic divergence from closely related taxa (e.g. *U. ohnoi* and *U. californica*). Overall, although the number of *Ulva* species groups identified by ABGD was dependent on P and X values, the results were largely congruent with those of the phylogenetic analyses. Accordingly, ABGD results were interpreted as complementary to phylogenetic inference, and ten major *Ulva* species groups were retained under the representative parameter set, based on *tuf*A (*P* = 0.0077, X = 1.0; Table 1; Table S4).

**Table 1:** The number of groups (clusters) obtained from the ABGD analysis using the chloroplast (*tuf*A) marker for *Ulva* samples from Jeju Island and thesouthern coasts.

Species identified by phylogenetic analyses (NJ, ML)	ABGD (based on *tuf*A)			
	X=1.0		X=1.5	
	P= 0.0077	P=0.0129	P=0.0077	P=0.0129
*U. australis ( =U. pertusa)*	2	2	2	2
*U. ohnoi*	1	1	1	1
*U. lactuca*	0	0	0	0
*U. californica*	1	1	1	1
*U. lacinulata*	1	0	0	0
*U. aragoënsis*	0	0	0	0
*U. arasakii*	1	1	1	1
*U. compressa*	1	1	1	1
LPP sp. 1(*U. procera*)	2	1	0	0
LPP sp. 2(*U. prolifera*)	1	1	0	0
*Ulva *sp.	5	5	5	5
Total number of groups (clusters)	15 groups	13 groups	11 groups	11groups
Total number of groups (clusters) of *Ulva* specimens analyzed in this study	10 groups	8 groups	6 group	6 group

### Species identification based on *tuf*A-based phylogenetic analysis

**Fig. 3:**
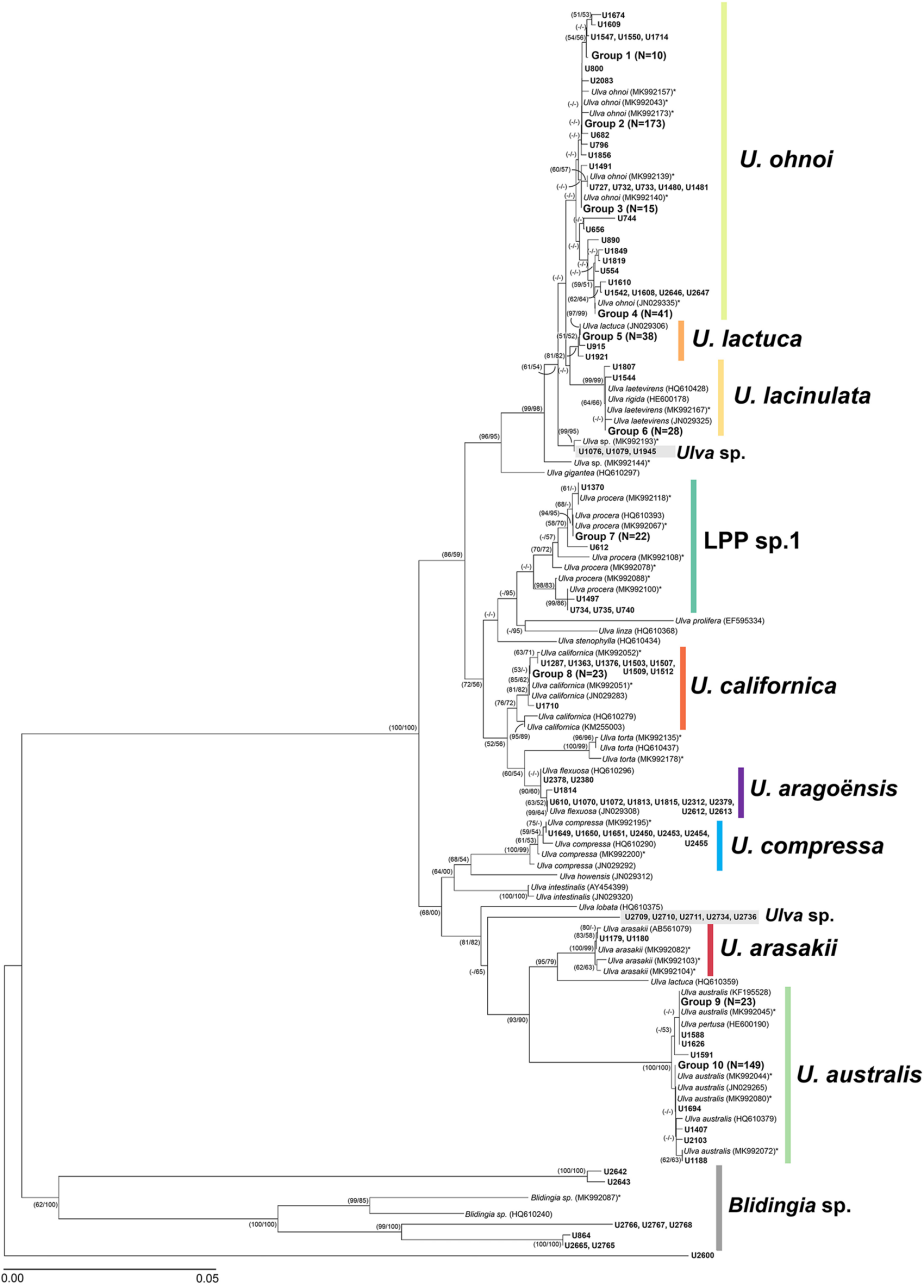
Neighbor-joining (NJ) phylogeny based on 669 *tuf*A sequences (612 specimens of Jeju Island plus 55 previously determined haplotype sequences and two unidentified haplotypes) of *Ulva* and two sequences of *Blidingia* species as outgroup^18^. Reference sequences were obtained from GenBank and used for phylogenetic analysis of species identification. Specimens analyzed in this study are highlighted in bold and labeled with U numbers. A large number of specimens found within a particular clade are marked as “Group” with the total number of specimens [e.g., Group 1 (*N* = 40)]. Numbers on the nodes indicate bootstrap values for maximum likelihood (ML) and neighbor-joining (NJ), respectively.

The *tuf*A phylogeny showed that in 612 specimens from Jeju Island, 266 individuals (43.46%) were identified as *U. ohnoi*, 179 (29.25%) as *U. australis* (= *U. pertusa*), 40 (6.54%) *Ulva lactuca*, 31 (5.07%) *U. californica*, 30 (4.90%) *U. lacinulata*, 28 (4.58%) belonging to the LPP sp. 1 (provisionally identified as *U. procera* based on *tuf*A), 12 (1.96%) *U. aragoënsis*, 7 (1.14%) *U. compressa*, 2 (0.33%) *U. arasakii*, and 8 (1.31%) *UIva* sp. *Blidingia* spp. (8 specimens) and *Gayralia* sp. (1 specimen) were also identified, other than the genus *Ulva* (Fig. 3). The analysis showed that a well-supported clade previously identified as *U. laetevirens* or *U. rigida* was conspecific with *U. lacinulata*, in accordance with recent taxonomic revisions based on type-derived sequence analyses^37^. Accordingly, this lineage was hereafter treated as *U. lacinulata* (syn. *U. laetevirens*, *U. armoricana*, *U. scandinavica*)^37^.

**Fig. 4:**
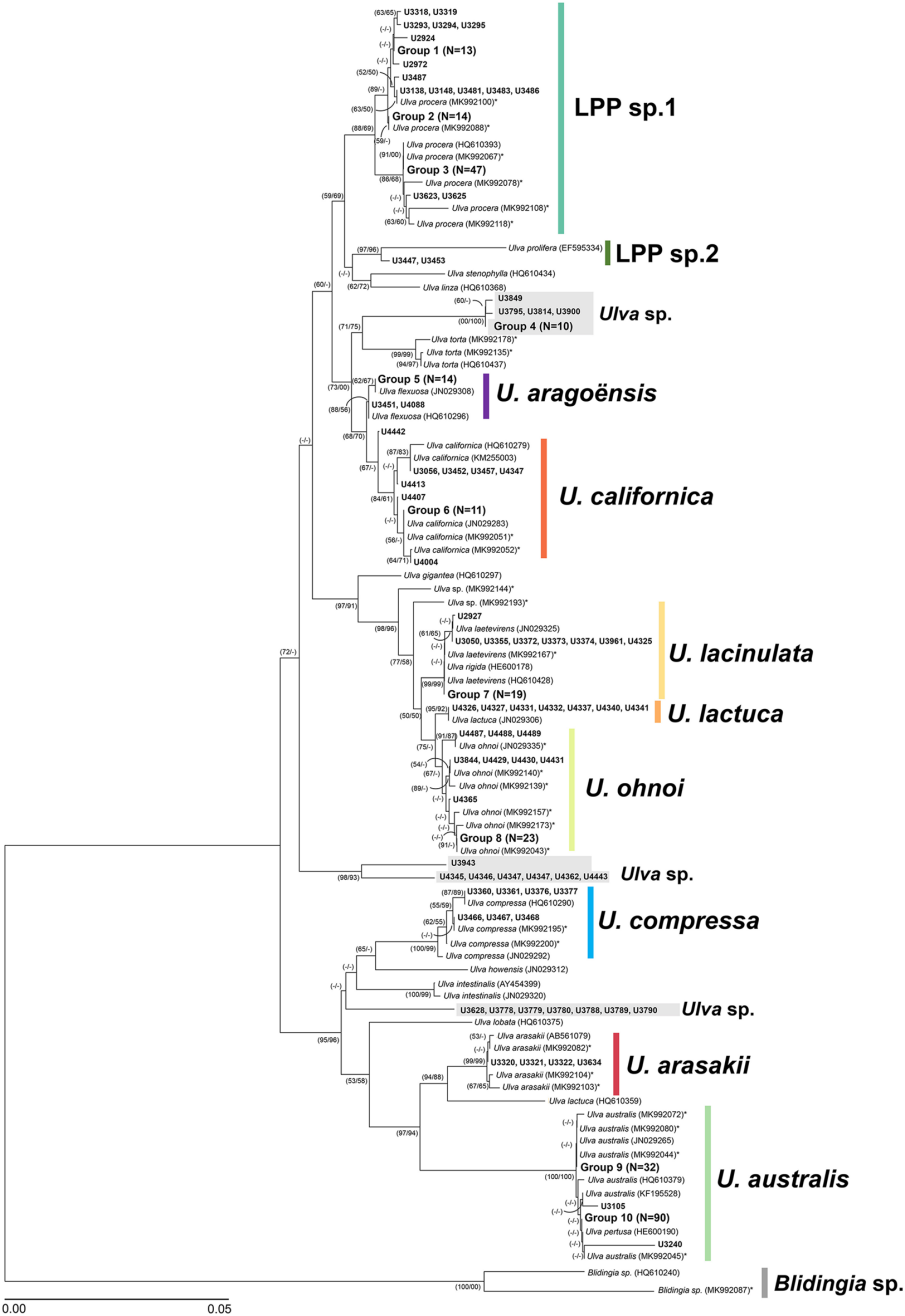
Neighbor-joining (NJ) phylogeny based on 411 *tuf*A sequences (354 specimens of the southern coasts plus 55 previously determined haplotype sequences and two unidentified haplotypes) of *Ulva* and two sequences of *Blidingia* species^18^. Reference sequences were obtained from GenBank and used for phylogenetic analysis of species identification. Specimens analyzed in this study are highlighted in bold and labeled with U numbers. A large number of specimens found within a particular clade are marked as “Group” with the total number of specimens [e.g., Group 1 (*N* = 13)]. Numbers on the nodes indicate bootstrap values for maximum likelihood (ML) and neighbor-joining (NJ), respectively.

By comparison, for a total of 354 specimens from the southern coasts, 123 individuals (34.75%) were determined as *U. australis*, 89 (25.14%) LPP sp. 1 (*U. procera*), 31 (8.76%) *U. ohnoi*, 28 (7.91%) *Ulva* sp., 27 (7.63%) *U. lacinulata*, 18 (5.08%) *Ulva californica*, 17 (4.80%) *U. aragoënsis*, 7 (1.98%) *U. lactuca*, 7 (1.98%) *U. compressa*, 4 (1.13%) *U. arasakii*, and 2 (0.56%) LPP sp. 2 (*U. prolifera*). Only a single specimen was identified as *Blidingia* sp. (Fig. 4). The phylogenetic tree encompassing the entire dataset from Jeju Island and the southern coasts is provided in Fig. S2. In the analyses, specimens belonging to Group 2 (Fig. 3), Group 8 (Fig. 4), and Group 12 (Fig. S2) within *U. ohnoi* clades clustered together with the reference sequences of *Ulva pseudo-ohnoi* described by a previous study^31^. These sequences showed 100% identity in the *tuf*A region and did not form a distinct lineage in our phylogenetic analysis. Therefore, *U. pseudo-ohnoi* could not be resolved as a separate clade from *U. ohnoi* based on the present *tuf*A dataset.

### The 5S rDNA analysis for *Ulva* species belonging to the LPP clade

**Fig. 5:**
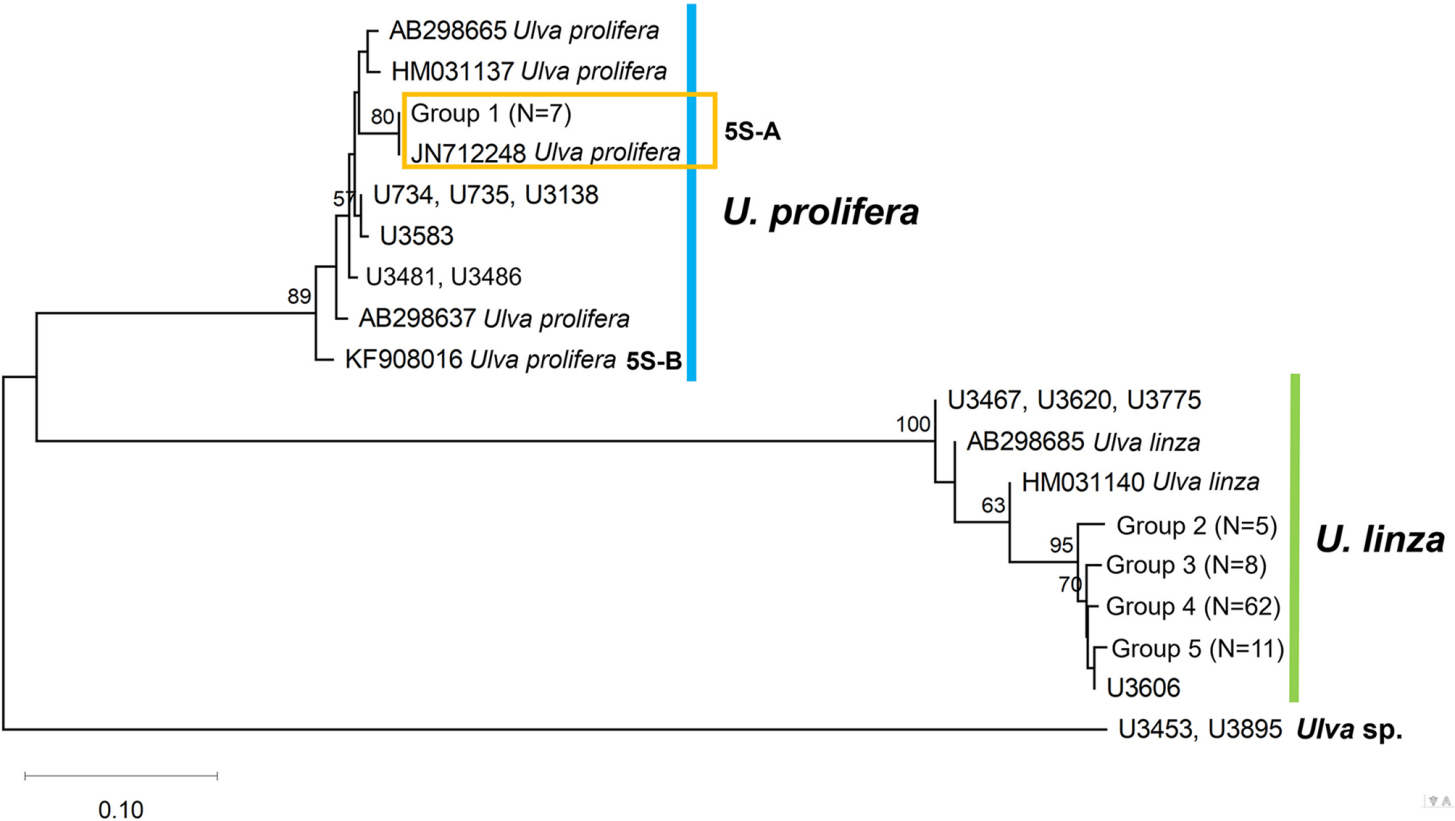
Unrooted neighbor-joining (NJ) tree of 5S rDNA spacer region of the LPP complex. Bootstrap values of ≥ 50% for 1,000 replicates are given at each node. Groups were defined when the same single lineage was formed.

**Fig. 6:**
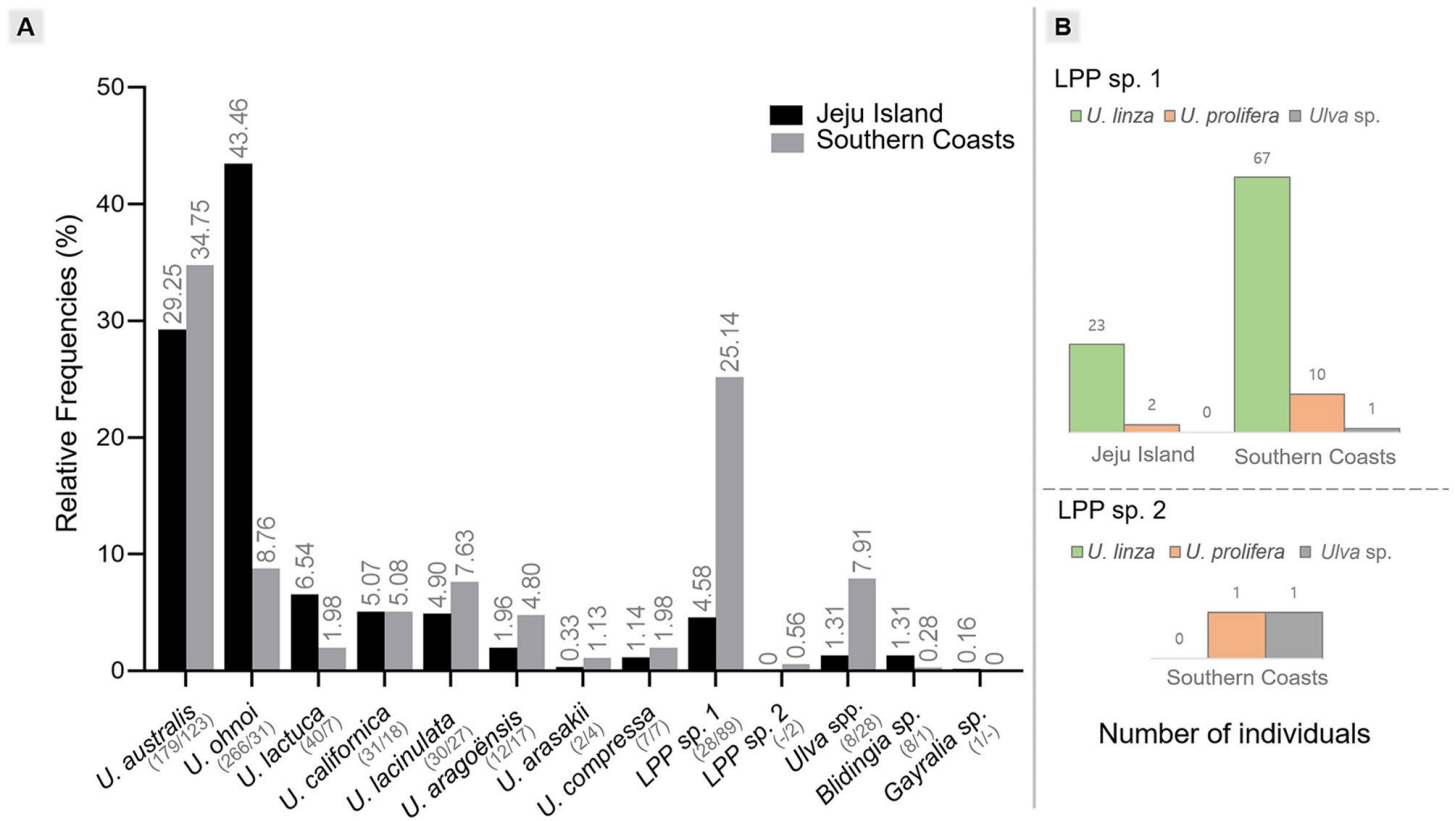
Comparisons of relative frequencies of *Ulva* species between *Ulva*-communities from Jeju Island and the southern coasts based on a combined analysis of *tuf*A (**A**) and 5 S rDNA (**B**). Jeju Island; samplings conducted from November 2019 to February 2021, the southern coasts; from January 2021 to October 2021. LPP sp. 1 was identified as *U. procera*, while LPP sp. 2 was identified as *U. prolifera*, based on *tuf*A analysis (**A**). Both LPP sp. 1 and LPP sp. 2 were subsequently reclassified into *U. linza*, *U. prolifera*, and *Ulva* sp., based on 5S rDNA (B).

The 5S rDNA analysis revealed that specimens initially assigned to *U. procera* based on the *tuf*A marker were separated into two major lineages corresponding to *U. linza* and *U. prolifera* (Fig. 5). Most specimens initially assigned as *U. procera* based on *tuf*A formed a distinct clade corresponding to *U. linza* (*N* = 90), whereas the remaining specimens grouped within the *U. prolifera* clade (*N* = 13), together with two *Ulva* sp. Samples (Fig. 6). Samples initially identified as *U. procera* by *tuf*A were re-classified into *U. linza* and *U. prolifera* groups, except for two individuals (U3453 originally assigned to LPP sp. 2, and U3895 LPP sp. 1) that did not cluster with either group (Fig. 6). Among the 25 samples from Jeju Island, 23 were identified as *U. linza* and 2 as *U. prolifera*. On the southern coasts, 67 individuals were identified as *U. linza*, 11 as *U. prolifera*, and 2 remained *Ulva* sp. (Figs. 5, 6B). All southern coast sites contained members of the LPP clade except for Baekya in Yeosu (Fig. 1; Table S2). In contrast, on Jeju Island, the LPP clade-species were detected only at four sites, including the Jongdal, inside Hanlim harbor, Hyeopjae, and inside Sinchang 1 (Fig. 1; Table S1). Within the *U. prolifera* lineage, all specimens obtained in this study clustered exclusively with the 5S-A reference sequences, and none grouped with the 5S-B lineage (Fig. 5), as indicated by a previous study^40^.

### Spatial variation in *Ulva* community structure between Jeju Island and the southern coasts

The relative frequencies, distributional patterns, species composition and diversity of the entire *Ulva* communities were compared between Jeju Island and southern coasts. By excluding *Ulva* sp. specimens, 9 *Ulva* species were identified for Jeju Island, whereas 10 species were found for the southern coasts, based on *tuf*A phylogenies (Fig. 6). However, when the 5S rDNA results were considered (Fig. 6B), a total of 11 species were identified from both Jeju Island and the southern coasts (Table 2).

For the relative frequencies of *Ulva* species in Jeju Island, two species, *U. ohnoi* (43.68%) and *U. australis* (29.39%), were the most predominant across all the geographic and seasonal samples (Table 2; Figs. 5 and 6). Besides, the Jeju Island-*Ulva* community was composed of *U. lactuca* (6.57%), *U. californica* (5.09%), *U. lacinulata* (4.93%), *U. linza* (3.78%), *U. aragoënsis* (1.97%), *Ulva* sp. (1.31%), *U. compressa* (1.15%), *U. arasakii* (0.33%) and *U. prolifera* (0.33%) in this order, based on the combined results of *tuf*A and 5S rDNA (Table 2). In addition to *Ulva* species, other species belonging to class Ulvophyceae, such as *Blidingia* (*N* = 8) and *Gayralia* (*N* = 1) spp. By comparison, on the southern coasts *Ulva-*community was comprised of *U. australis* (35.86%), *U. linza* (19.53%), *U. ohnoi* (9.04%), *Ulva* sp. (8.75%), *U. lacinulata* (7.87%), *U. californica* (5.25%), *U. aragoënsis* (4.96%), *U. prolifera* (3.21%), *U. lactuca* (2.04%), *U. compressa* (2.04%) and *U. arasakii* (1.17%) in this order (Table 2; Fig. 6). In addition to *Ulva* species, other species belonging to class Ulvophyceae, such as *Blidingia* (*N* = 1) were also detected.

**Table 2:** Comparisons of relative frequencies of *Ulva* species communities between Jeju Island and the southern coasts, based on the combined results of *tuf*A-and 5 S rDNA-based phylogenetic analyses.

Species	Jeju Island		Southern Coasts	
	Sample size (*N*)	Relative frequency (%)	Sample size (*N*)	Relative frequency (%)
*U. australis*	179	29.39	123	35.86
*U. ohnoi*	266	43.68	31	9.04
*U. lactuca*	40	6.57	7	2.04
*U. californica*	31	5.09	18	5.25
*U. lacinulata*	30	4.93	27	7.87
*U. aragoënsis*	12	1.97	17	4.96
*U. arasakii*	2	0.33	4	1.17
*U. compressa*	7	1.15	7	2.04
U. linza*	23	3.78	67	19.53
U. prolifera*	2	0.33	11	3.21
*Ulva* spp.	8	1.31	30	8.75
*Blidingia* sp.	8	1.31	1	0.29
*Gayralia* sp.	1	0.16	-	-
Total	609		343	

*: LPP clade individuals not identified by 5S rDNA were excluded (Jeju Island: 3; Southern Coasts: 11).

**Fig. 7:**
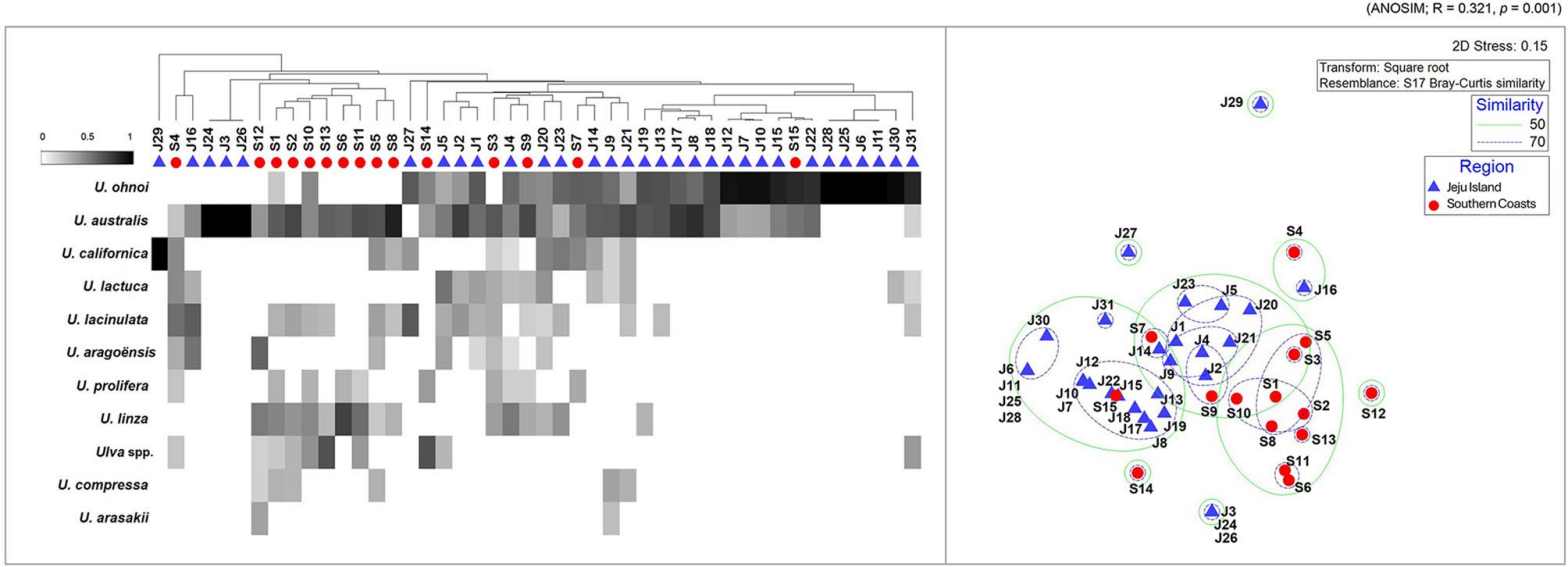
Results of community similarity analysis of *Ulva* species composition for Jeju Island and southern coasts, based on the combined results of *tuf*A-and 5S rDNA-based analyses. (**A**) Results of heat map analysis. (**B**) Results of non-metric multidimensional scaling (NMDS) plot analysis.

Bray–Curtis similarity analysis revealed a significant spatial difference in *Ulva* community structure between Jeju Island and the southern coasts (ANOSIM, *r* = 0.321, *p* = 0.001). In the NMDS ordination, samples from Jeju Island and the southern coasts clearly formed separate clusters, indicating distinct spatial segregation in *Ulva* community composition (Fig. 7). A few sites from the southern coasts overlapped with the Jeju Island cluster [S7 (Yegye 3), S9 (Sulcheon), and S15 (Baekya)], suggesting partial similarity in species composition between certain locations of the two regions.

Differences in life forms between *U. ohnoi* and *U. australis* were also apparent, with *U. ohnoi* exhibiting a higher proportion of floating thalli than *U. australis* on Jeju Island (Fig. S3). A chi-square analysis revealed a significant difference in the proportions of benthic and floating individuals between the two species (χ² = 16.51, *p* < 0.001). The within-species preference index (PI), calculated as (Floating − Benthic)/(Floating + Benthic), indicated that *U. ohnoi* had a strong tendency toward the floating form (PI = 0.48), whereas *U. australis* displayed nearly equal proportions of the two forms (PI = 0.04). When standardized using Ivlev’s^41^ and Jacobs’s^39^ electivity indices, *U. ohnoi* still showed a positive bias toward the floating form (E = 0.06; D = 0.20), while *U. australis* exhibited a weak preference for the benthic form (E = − 0.12; D = − 0.28).

###  Seasonal variation in *Ulva* community structure in Jeju Island and the southern coasts

**Fig. 8:**
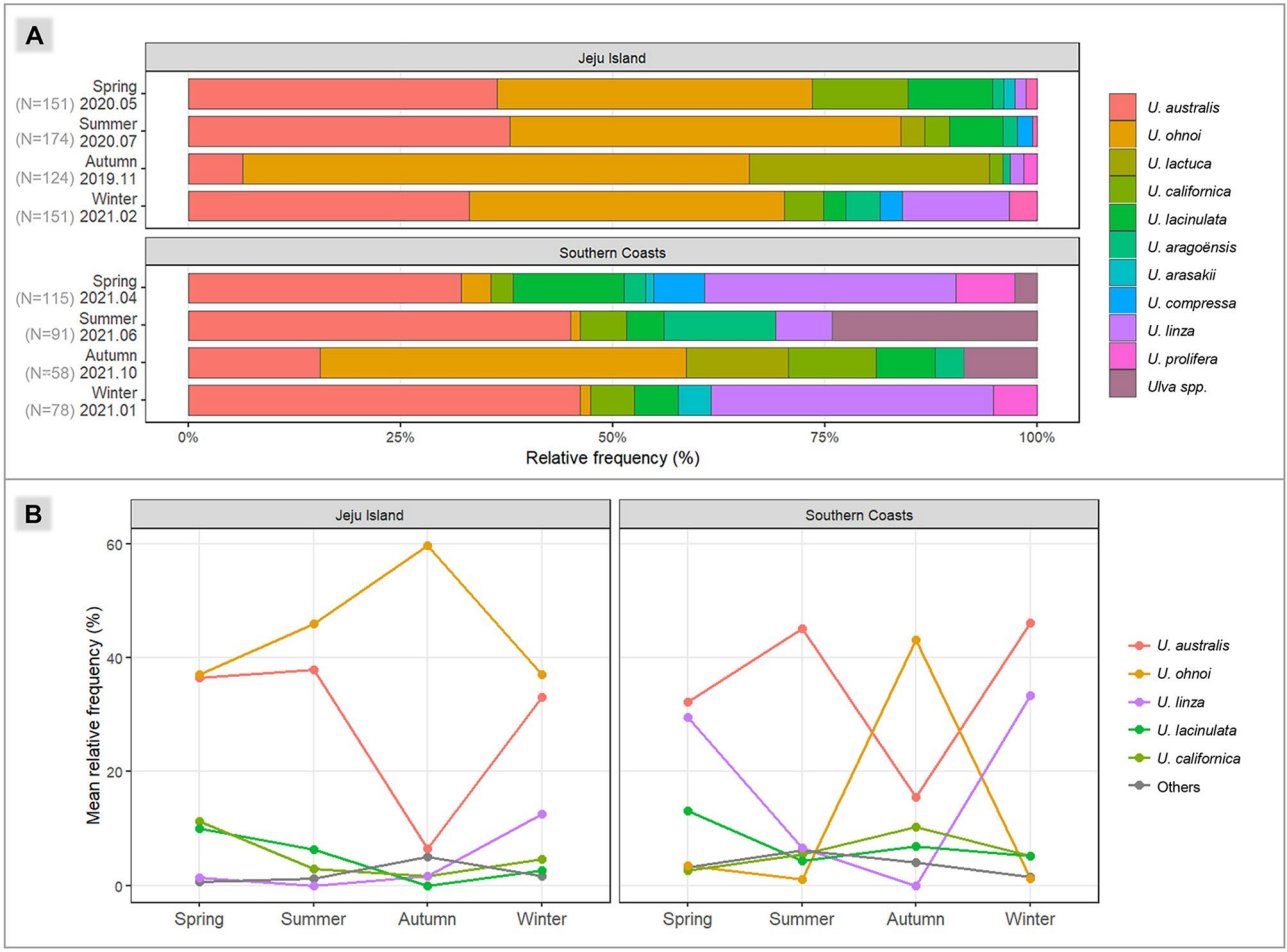
Seasonal changes in relative frequency (%) of *Ulva* species in green tide-forming assemblages in Jeju Island and the southern coasts across four seasons (spring, summer, autumn, and winter), based on the combined results of *tuf*A and 5S rDNA analyses. (**A**) Stacked bar charts showing the relative frequency of each species; species names are listed on the right. (**B**) Line graphs illustrating seasonal patterns of relative frequency (%) of the five dominant species in Jeju Island and the southern coasts.

Seasonal variation in the *Ulva* community structure, species composition and relative frequencies was examined for both geographic regions (Fig. 8). For Jeju Island, a total of 11 species were identified. Two species, *U. ohnoi* (37.09–59.68%) and *U. australis* (6.45–37.93%) were predominant all year round, regardless of the season (Fig. 8; Table S5). An average relative frequency of *U. ohnoi* and *U. australis* were 44.33%, 29.83%, respectively. While *U. ohnoi* exhibited a peak in relative frequency (59.68%) in autumn, *U. australis* showed the lowest frequency (6.45%) (Table S5). In addition, *U. lactuca* increased its frequency in autumn (28.23%) relative to other seasons (2.87% in summer and zero in winter and spring) (Fig. 8; Table S5). The other *Ulva* species also exhibited some seasonal variation in their frequencies. *Ulva californica* ranged from 1.61% to 11.26% (the highest frequency in spring), *U. lacinulata* 0.00%–9.93% (the highest in spring and absent in autumn), and *U. aragoënsis* 0.81%–3.97% (the highest in winter). *Ulva arasakii* was detected only in spring (1.32%). *Ulva compressa* appeared only in summer and winter, with a relative frequency ranging from 1.71% to 2.65% and *U. linza* 0.00%– 12.58% (the highest in winter), (Fig. 8; Table S5).

Similar to Jeju Island, on the southern coasts *Ulva* community structure also changed according to the season. *Ulva australis* (15.52–46.15%) and *U. linza* (0.00–33.33%) were the most dominant species, although *U. procera* was not present at all in autumn. *Ulva ohnoi* was low in its frequency (1.10–3.48%) except for during autumn with the highest peak (43.1%) (Fig. 8; Table S6). *Ulva lacinulata* indicated relatively stable frequencies across seasons (4.4%–13.04%) and *U. aragoënsis* showed a higher frequency in summer (13.19%) compared to the other seasons. *Ulva arasakii* exhibited a relative frequency of 3.85% during winter, which decreased to 0.87% in spring. *Ulva compressa* only occurred in spring (6.09%). *Ulva prolifera* was present (5.13–6.96%) only in spring and winter (Fig. 8; Table S6). These findings suggest that there was considerable seasonal variation in the species composition, distribution and relative frequencies between the two geographic regions.

## Discussion

Based on the exhaustive phylogenetic analysis on the spatial and seasonal samples of a total of 966 *Ulva* specimens from 46 sites along the coastlines of Jeju Island and the southern coasts of Korea, we find considerable differences in the species composition, diversity, spatial distribution and relative frequencies of the green tide forming *Ulva* communities between the two regions. In Jeju Island, the most significantly contributing species to local green tides turned out to be *U. ohnoi* and *U. australis*, which is consistent with the previous findings^16^^[,18^^[,31^. The dominance of these species in Jeju Island can be partially attributed to relatively higher sea surface temperature (SST; 14.76–15.83 °C) in this region (Fig. S1), which might provide favorable growth conditions for subtropical species like *U. ohnoi*. On the other hand, in the southern coasts of the mainland, *U. australis* and LPP clade species (e.g. *U. linza*) are predominant in green tide forming *Ulva* communities throughout the year. The higher concentrations of nitrogen compounds and chemical oxygen demand (COD) in the southern coasts create a nutrient-rich environment with a high nitrogen availability that supports the growth of these species (Fig. S1), although the green tide algal biomass is much lower relative to Jeju Island^29^. Although these findings suggest regional differences in environmental context, further investigation is required to clarify the ecological implications of individual environmental parameters more precisely. Seasonal variation in *Ulva* community structure was assessed based on biological sampling; however, seasonal environmental drivers could not be explicitly analyzed because environmental data were not available at comparable temporal resolution between the regions and also across the sites. Future studies integrating long-term, seasonally resolved environmental datasets will be necessary to directly link *Ulva* bloom dynamics with environmental forcing^42^.

*Ulva ohnoi*, the most common species in Jeju Island, is known to have a subtropical or tropical origin^43^. It was first identified and taxonomically classified in 2004 in Japan^43^ and is considered one of the major species responsible for green tides in several regions including Japan^44^^[,45^. Moreover, it has been reported as a widely distributed species in various areas, including South Africa^46^. Although several molecular studies have investigated *Ulva* species in Korean waters, particularly around Jeju Island^16^^[,18^^[,31^ the biogeographical origin of *U. ohnoi* remains unresolved. In particular, it is still unclear whether *U. ohnoi* populations observed along the Korean coasts represent long-established native lineages or more recent expansions from adjacent regions such as Japan or other subtropical areas. Additional analyses using existing sequence datasets from GenBank could help to clarify the phylogeographic structure and potential dispersal history of this species. To our knowledge, this study provides the first report of *U. ohnoi* occurrence along the southern coasts of the Korean Peninsula. Given the potential influence of subtropical climatic conditions in this region, it remains uncertain whether the observed populations represent recent range expansion or long-term establishment.

We also find differences in species composition, distribution and relative frequencies in both coastal regions according to the season. For Jeju Island, the primary contributors to the year-round local green tides were *U. ohnoi* and *U. australis*, which tend to dominate the *Ulva* community regardless of the season, except *U. australis* was replaced with *U. lactuca* in autumn. The two species, *U. ohnoi* and *U. australis*, are known to be remarkably tolerant to high temperatures, which may contribute to sustaining large amount of green macroalgal biomass even in summer/autumn. On the other hand, for the southern coasts, *U. ohnoi* shows a rapid increase in frequency only during autumn. It has been reported that *U. australis* shows a high growth rate in spring and early summer but tends to decline sharply in August^47^. It would therefore be conceivable that the relative frequency of *U. ohnoi* increased rapidly in autumn due to the decrease in *U. australis*. This seasonal pattern may also reflect differences in species-specific thermal optima and temperature preferences between the two species^48^. Still, *U. ohnoi* was rarely seen in other seasons (but spring), and *U. linza* species occurred in every season except for autumn.

The dominance of *U. linza* on the southern coasts may be attributed to several factors. This species is well-known for its physiological adaptability and tolerance to varying environmental conditions, which may confer a competitive advantage in this region^49^. Furthermore, *U. linza* has been reported to withstand fluctuations in temperature and salinity^49^, likely contributing to its year-round presence along the southern coasts.

*Ulva australis* is a widespread intertidal species that occurs along the entire coastline of Japan^50^. It is known to cause green tide outbreaks in temperate regions of southern and western Japan, along the Pacific coast of central Japan^43^^[,51^. This species also maintains relatively high biomass during winter^30^^[,50^ and forms extensive green tides in various parts of the world, including the northwestern Atlantic coast of the Iberian Peninsula (Galicia, Spain)^52^ and the northern coasts of China^53^. According to Hanyuda and Kawai^54^, *Ulva pertusa* Areschoug, 1851 and *Ulva australis* Kjellman, 1897 were suggested as synonym, which was supported by our previous phylogenetic analyses demonstrating the monophyly of these taxa^16^^[,18^. This species is among the most common causes of green tides in Korea and Japan, with its dominance particularly well documented in Jeju Island from 2015 to 2020^16^.

A recent study suggests that *U. ohnoi* has the potential to undergo explosive growth in high-temperature marine ecosystems driven by ocean warming and acidification^55^. This subtropical to tropical species exhibits a high growth rate even under elevated summer water temperatures^51^. As one of the most abundant and widely distributed *Ulva* species, *U. ohnoi* is expected to cause green tides more frequently and severely under warmer environmental conditions^45^^[,48^. Temperature plays a crucial role in promoting photosynthesis and growth of *U. ohnoi*, as higher temperatures enhance the growth of both young and adult thalli by increasing metabolic activity, particularly during summer. According to Korea Oceanographic Data Center from the National Institute of Fisheries Science (NIFS, Korea; https://www.nifs.go.kr/kodc/) in Korea, a rise in SST was evident for the last five years (from 2015 to 2020 in April for Jeju Island). In 2015, the average SST across the four stations was 14.70 °C (± 1.42 °C SD). In 2020, the average SST increased to 15.61 °C (± 1.51 °C SD). These values clearly demonstrate a noticeable increase in SST from 2015 to 2020, with all stations showing an upward trend. This temperature increase is noteworthy as it creates favorable growth conditions for *U. ohnoi*^16^^[,56^.

*U. ohnoi* primarily grows attached to rocky substrates, but detached floating fronds can also proliferate vegetatively by rapidly absorbing nutrients. The observed high proportions of floating-thalli form of *U. ohnoi* (Fig. S3) can provide evidence supporting this hypothesis. This trait contributes to frequent green tide outbreaks in coastal areas^29^^[,51^. In Japan, *U. ohnoi* is widely distributed on the southwest coast, where the sea temperature is warm, whereas *U. australis* is predominant on the northeast coast^48^.

Several *Ulva* species detected in this study (e.g. LPP-clade species, *U. aragoënsis*, and *U. californica*) have been reported as non-native or introduced in some regions, often in association with shipping-related vectors^57^. Nonetheless, their native vs. introduced status in Korean waters remains to be tested. In particular, the reported high genetic variability in LPP species in East Asia may suggest the possibility that these taxa may have originated or diversified in this region^22^. *Ulva californica*, a species native to the Pacific Coast of North America, has recently been reported to be introduced to Europe, including Ireland and the United Kingdom, the Mediterranean, Oceania, and also Asia by hull for maritime transportation^58^^[,59^^[,60^. This species was first recorded from California (type locality: La Jolla, Collins et al., 1899: no. 611) and has never been reported from the Mediterranean until 2012^59^. It has physiological and ecological capabilities that enable to survive and grow even in harsh environmental conditions such as a lack of light for more than 10 months^61^. *Ulva californica* was shown to cause green tide events in Chile and California, USA^61^. Notably, although *U. californica* was present in other areas, a particular site (Population ID 29; Seogwipo harbor) shows only *U. californica*. This finding suggests that maritime transport and harbor environments may facilitate the establishment of species such as *U. californica*, although direct evidence for introduction pathways is not available in the present study. Such unique occurrences underscore the importance of monitoring harbor environments where nonindigenous species may establish and proliferate after successful colonization. In addition, *U. linza* and *U. aragoënsis* are often reported as the potentially problematic species causing macroalgal blooms, and there is also a possibility of causing green tides in Korea in the future^57^^[,62^^[,63^. Fortunately, *U. californica* and *U. lacinulata*, which have been reported as introduced or cryptogenic in some regions, they were not major contributors to present-day green tide events along the Korean coast, as suggested by our results. Nevertheless, continuous observation and monitoring are required, as these species have been identified as major contributors to green tide events in various parts of the world^64^.

Comparison of our *tuf*A sequences with all publicly available NCBI records revealed that the *U. aragoënsis*-related lineage detected in this study is not restricted to East Asia. Several of our sequences showed 100% identity to reference sequences originating from Europe, North America, and other regions. Among our samples, most sequences from Jeju Island (*N* = 9) and southern coasts (*N* = 13), with the exception of three samples (U2378, U2380, and U1814) from Jeju Island and two (U3451 and U4088) from the southern coasts, were identical to reference sequences previously identified as *U. mediterranea* and later reclassified as *U. aragoënsis*^65,66,]^^67^. These findings indicate that multiple *tuf*A lineages related to *U. aragoënsis* are present in Korean waters.

*Ulva lacinulata* (historically reported under the names *U. laetevirens* and *U. rigida*) was first collected in Australia in 1854 and has been reported in several Mediterranean countries since the late 1990s^68^. This species exhibits an optimal growth rate at temperatures between 12 °C and 23 °C^64^. During summer, its growth declines when temperatures exceed these optimal values^15^^[,64^ as ambient temperature is a critical factor affecting its development and growth. Consequently, the relative frequency of *U. lacinulata* on the southern coasts remained relatively stable or even slightly higher during mild temperature periods such as spring and autumn, compared with summer and winter.

*Ulva prolifera*, one of the three species (*U. linza* and *U. procera* being the other) belonging to the LPP clade, is notorious for causing massive macroalgae blooms, green tide events in the Yellow Sea of China^26,69^. Although *U. prolifera* is a cosmopolitan species, large-scale green tides in the Yellow Sea have been mainly associated with specific ecological populations linked to coastal aquaculture areas in China^70,71^. In the present study, both *U. linza* (*N* = 67; 85.90%) and *U. prolifera* (*N* = 10; 12.82%) were identified in the southern coast region based on 5S rDNA analysis. On Jeju Island, *U. linza* (*N* = 23; 92%) and *U. prolifera* (*N* = 2; 8%) were also detected. These findings might suggest that *U. prolifera* observed along the southern coasts of Korea may have been introduced from the east coast of China^72^. However, more solid lines of evidence (e.g. population genetics/genomics analysis among Chinese, Korean, and European *U. prolifera* samples) are required before the conclusions about introduction pathways. Further research will help verify this hypothesis and determine whether *U. prolifera* populations in Korean waters originated through recent introductions or natural dispersal^71^.

Based on 5S rDNA analysis, *U. linza* occurs in both Jeju Island and the southern coasts. On Jeju Island, it occurred at four of 31 sites (12.90% of all sites), with relative abundances ranging from 3.23% (inside Hallim harbor and inside Sinchang 1) to 58.06% (Jongdal). On the southern coasts, *U. linza* was present at 14 of 15 sites, ranging from 1.49% (Sagok) to 14.92% (Deokho). Moreover, species belonging to the LPP clade were widely distributed along the entire southern coastal region, whereas their occurrence on Jeju Island was limited to four sites: inside Hallim Harbor, inside Sinchang 1, Jongdal, and Hyeopjae. These findings provide important insights into the species composition and distributional patterns of *Ulva* communities in the studied coastal regions.

For the LPP clade, previous studies have shown that neither *tuf*A nor ITS markers can reliably differentiate among the three species (*Ulva linza-procera-prolifera*)^22^. Therefore, in this study, the 5S rDNA marker was analyzed to achieve more accurate species identification within the LPP clade^22^^[,24^. The analysis targeted the specimens classified as members of the LPP clade based on *tuf*A-derived phylogenetic results. The 5S rDNA spacer region, which has been demonstrated to resolve interspecific relationships within *Ulva* was used here for unambiguous species identification within the LPP clade^25^^[,26^^[,73^^[,74^. Previous studies have demonstrated that *U. prolifera* can be subdivided into two major 5S rDNA spacer types, 5S-A (also referred to as 5S-I) and 5S-B (5S-II)^40^^[,75^^[,76^. In particular, 5S-B has been reported to replace 5S-A as the dominant lineage in later stages of persistent green tides, suggesting ecological or physiological differences between the two types. In the present study, eight out of the 14 *U. prolifera* specimens clustered exclusively with the 5S-A reference lineage, whereas the 5S-B lineage was not detected (Fig. 5). This pattern indicates that the *U. prolifera* populations observed along the Korean coasts during the study period may represent an early-stage or non-blooming lineage, rather than the lineage associated with massive and persistent green tides in the Yellow Sea. Nevertheless, the presence of 5S-A suggests a potential risk of future large-scale bloom development, highlighting the importance of continuous monitoring. Furthermore, recent taxonomic revisions have proposed a reinterpretation of species boundaries within the LPP clade. Specifically, a very recent study suggested that the LPP clade consists of *U. smaragdina* and *U. prolifera*, based on genomic analyses of type specimens^77^. This framework implies that lineages previously identified as *U. linza* and *U. procera* may correspond to *U. smaragdina* and *U. prolifera*, respectively. However, although this revision is supported by extensive genomic data derived from type material, its applicability to globally distributed *Ulva* lineages remains to be confirmed.

Together with earlier taxonomic work and biodiversity records, this study provides further evidence for the high species diversity of *Ulva* in Korean waters. Our genetic analyses revealed a greater extent of hidden diversity with *Ulva* communities in Jeju Island and the southern coasts of Korea, indicating that the actual number of species has likely been underestimated. In particular, specimens previously identified as *U. procera* were re-identified as *U. prolifera* and *U. linza* based on 5S rDNA analysis, highlighting that the limited resolution of certain markers may lead to an underestimation of *Ulva* species diversity. For example, although *U. prolifera* and *U. linza* are genetically distinct, earlier morphological studies might have misidentified one as the other due to phenotypic plasticity and overlapping morphological features. In addition, some *Ulva* taxa previously reported from Korean waters, such as *Ulva torta*, were not detected in the present study, suggesting that our species inventory may not fully capture the complete *Ulva* diversity along the entire Korean coast^78^. Therefore, further molecular surveys across additional localities with increasing sampling efforts will be required to achieve a more comprehensive inventory of *Ulva* species throughout the Korean coast. Our findings emphasize the importance of accurate species identification using molecular approaches to avoid taxonomic confusion and improve our understanding of *Ulva* biodiversity. Identifying the dominant *Ulva* species responsible for local green tide events and elucidating their physiological and ecological characteristics are critical steps toward developing effective management strategies to reduce bloom occurrences.

In this study, we applied model-based species delimitation in conjunction with molecular phylogenetic analyses to enhance the precision of species identification. The resulting molecular data provide a valuable baseline for understanding the genetic structure and community composition of green tide–forming *Ulva* species along the coasts of Jeju Island and the southern coasts, and for supporting continuous monitoring and management of bloom-forming and potentially non-native species.

## Conclusion

This study provides an in-depth assessment of spatial and seasonal variations in *Ulva* species composition and diversity along the coasts of Jeju Island and the southern coasts of Korea, highlighting their contributions to green tide formation. Phylogenetic analyses using *tufA* and 5S rDNA markers revealed distinct *Ulva* community structures between the two regions. *U. ohnoi* and *U. australis* predominated on Jeju Island, whereas *U. linza* and *U. australis* were dominant along the southern coasts. These patterns suggest that environmental factors—such as temperature, nutrient availability, and species-specific ecological traits—drive regional and seasonal differences in *Ulva* communities, despite the need of future research. The dominance of *U. ohnoi* on Jeju Island highlights its potential to expand under warming conditions, increasing the risk of more frequent and severe green tides. Likewise, the prevalence of *U. linza* on the southern coasts indicates its resilience to environmental fluctuations. The detection of potentially nonindigenous species, including *U. californica*, and *U. aragoënsis*, underscores the need for continued genetic monitoring of *Ulva* populations. Our findings provide essential baseline data for developing targeted management strategies to mitigate the ecological and economic impacts of green tides in Korean coastal waters. Future research should investigate the ecological and physiological characteristics of dominant *Ulva* species and their responses to changing environmental conditions, and also their interactions with other coexisting species within the ecosystem.

## Supplementary Information

Below is the link to the electronic supplementary material.

Supplementary Material 1

## Data Availability

All sequence data generated in this study have been deposited in GenBank under accession numbers PZ285994–PZ286088 and are publicly available through the NCBI nucleotide database (https://www.ncbi.nlm.nih.gov/nuccore/). All other data supporting the findings of this study are available from the corresponding author upon reasonable request.
