# Supplementary material for: Molecular identification of major green tide-forming Ulva species and their spatiotemporal patterns on the Korean coast
Source: Sci Rep. 2026 May 5;16:20678. doi: 10.1038/s41598-026-50151-8 (PMC13333886; doi:10.1038/s41598-026-50151-8)

**Supplementary** **material**

**Table S1** Detailed geographic information of sampling sites of *Ulva* species along the coasts of Jeju Island, Korea between November 2019 and February 2021. *Ulva* specimens were collected during every season and thus they were sampled from every site four times. Sample size denotes the number of specimens sequenced for the *tuf*A (total *N*=612).

| **Population ID** | **Sampling site** | **latitude** | **longitude** | **Sample size (n)** |
| --- | --- | --- | --- | --- |
| 1 | Sinyang | 33°26'5.30"N | 126°55'22.79"E | 43 |
| 2 | Ojo (Seongsan harbor) | 33°28'13.87"N | 126°55'20.90"E | 30 |
| 3 | Tongbadal | 33°28'10.65"N | 126°55'24.51"E | 3 |
| 4 | Jongdal | 33°28'22.10"N | 126°54'45.15"E | 61 |
| 5 | Jongdal (cultivation) | 33°29'58.93"N | 126°54'40.82"E | 37 |
| 6 | Hado | 33°30'42.01"N | 126°53'53.60"E | 15 |
| 7 | inside Tokki-seom | 33°31'17.25"N | 126°53'59.70"E | 17 |
| 8 | outside Tokki-seom | 33°31'18.58"N | 126°54'0.29"E | 18 |
| 9 | Gimnyeong | 33°33'28.36"N | 126°45'29.69"E | 31 |
| 10 | Gimnyeong harbor | 33°33'18.01"N | 126°44'17.19"E | 16 |
| 11 | Hamdeok | 33°32'43.97"N | 126°39'47.93"E | 9 |
| 12 | inside Hamdeok | 33°32'53.75"N | 126°39'20.21"E | 21 |
| 13 | outside Hamdeok | 33°32'54.79"N | 126°39'22.53"E | 28 |
| 14 | Bangsatab | 33°32'58.91"N | 126°38'51.04"E | 39 |
| 15 | Jocheon | 33°32'19.38"N | 126°38'4.48"E | 12 |
| 16 | Iho | 33°29'52.30"N | 126°27'7.23"E | 10 |
| 17 | Aewol harbor | 33°28'2.89"N | 126°19'13.77"E | 5 |
| 18 | outside Hallim harbor | 33°25'22.33"N | 126°15'46.20"E | 6 |
| 19 | inside Hallim harbor | 33°25'22.01"N | 126°15'45.95"E | 24 |
| 20 | Hyeopjae | 33°23'43.36"N | 126°14'24.36"E | 34 |
| 21 | Geumneung | 33°23'23.61"N | 126°14'6.44"E | 34 |
| 22 | Sincnang 1 | 33°20'37.41"N | 126°10'29.09"E | 15 |
| 23 | inside Sincnang 1 | 33°20'46.60"N | 126°10'46.02"E | 12 |
| 24 | outside Sinchang 1 | 33°20'47.24"N | 126°10'41.69"E | 4 |
| 25 | Sinchang 2 | 33°20'35.00"N | 126°10'18.05"E | 8 |
| 26 | Moseulpo harbor | 33°13'9.00"N | 126°14'59.50"E | 9 |
| 27 | Hwasun | 33°14'23.27"N | 126°19'58.08"E | 12 |
| 28 | Daepyeong | 33°14'13.56"N | 126°21'42.02"E | 7 |
| 29 | Seogwipo harbor | 33°14'21.34"N | 126°33'31.84"E | 8 |
| 30 | Wimi | 33°16'8.94"N | 126°39'20.34"E | 12 |
| 31 | Pyoseon | 33°18'34.58"N | 126°49'52.98"E | 32 |

**Table S2** Detailed geographic information of sampling sites of *Ulva* species along the southern coastal area (the South Sea) of Korea in January 2021 - October 2021. *Ulva* specimens were collected during every season and thus they were sampled from every site four times. Sample size denotes the number of specimens sequenced for the *tuf*A (total *N*=354).

| **Population ID** | **Sampling site** | **latitude** | **longitude** | **Sample size (N)** |
| --- | --- | --- | --- | --- |
| 1 | Daldo 1 | 34°23'33.58"N | 126°38'33.82"E | 25 |
| 2 | Daldo 2 | 34°23'29.46"N | 126°38'31.70"E | 24 |
| 3 | Bulmok | 34°23'46.15"N | 126°42'15.03"E | 29 |
| 4 | Daeya | 34°21'54.30"N | 126°43'51.04"E | 22 |
| 5 | Yegye 1 | 34°48'58.54"N | 127°49'41.62"E | 23 |
| 6 | Yegye 2 | 34°48'59.66"N | 127°49'40.75"E | 11 |
| 7 | Yegye 3 | 34°49'18.40"N | 127°49'18.14"E | 22 |
| 8 | Chamyeon | 34°54'49.64"N | 127°51'0.35"E | 37 |
| 9 | Sulcheon | 34°56'45.61"N | 127°53'37.63"E | 27 |
| 10 | Gyeonyu | 34°53'9.48"N | 128°28'10.45"E | 38 |
| 11 | Deokho | 34°52'52.72"N | 128°28'33.26"E | 28 |
| 12 | Daegeum | 34°58'15.60"N | 128°41'58.23"E | 32 |
| 13 | Sagok | 34°53'59.00"N | 128°34'21.36"E | 13 |
| 14 | Hitdo | 34°37'50.46"N | 127°38'9.47"E | 13 |
| 15 | Baekya | 34°37'11.95"N | 127°38'28.71"E | 10 |

**Table S3** Detailed information on species name, GenBank accession number and relevant references for the 57 *tuf*A DNA sequences used for the molecular phylogenetic analyses for *Ulva* species identification (Fig. 3, 4).

| *Ulva* Species | Accession no. | reference | *Ulva* Species | Accession no. | reference |
| --- | --- | --- | --- | --- | --- |
| *Ulva ohnoi* | JN029335 | Kirkendale et al. (2013) | *Ulva compressa* | HQ610290 | Saunders and Kucera (2010) |
|  | MK992043 | Kang et al. (2019) |  | MK992195 | Kang et al. (2019) |
|  | MK992140 | Kang et al. (2019) |  | MK992200 | Kang et al. (2019) |
|  | MK992173 | Kang et al. (2019) |  | JN029292 | Kirkendale et al. (2013) |
|  | MK992157 | Kang et al. (2019) | *Ulva howensis* | JN029312 | Kirkendale et al. (2013) |
|  | MK992139 | Kang et al. (2019) | *Ulva intestinalis* | JN029320 | Kirkendale et al. (2013) |
| *Ulva lactuca* | JN029306 | Kirkendale et al. ( 2013) |  | AY454399 | O'Kelly et al. (2004) |
|  | HQ610359 | Saunders and Kucera (2010) | *Ulva arasakii* | AB561079 | Matsumoto et al.( 2011) |
| *Ulva lacinulata* | HQ610428 | Saunders and Kucera (2010) |  | MK992082 | Kang et al. (2019) |
|  | HE600178 | Wolf et al. (2012) |  | MK992103 | Kang et al. (2019) |
|  | JN029325 | Kirkendale et al. (2013) |  | MK992104 | Kang et al. (2019) |
|  | MK992167 | Kang et al. (2019) | *Ulva australis (=U. pertusa)* | MK992044 | Kang et al. (2019) |
| *Ulva gigantea* | HQ610297 | Saunders and Kucera (2010) |  | MK992080 | Kang et al. (2019) |
| *Ulva californica* | MK992051 | Kang et al. (2019) |  | MK992072 | Kang et al. (2019) |
|  | MK992052 | Kang et al. (2019) |  | MK992045 | Kang et al. (2019) |
|  | JN029283 | Kirkendale et al. (2013) |  | JN029265 | Kirkendale et al. (2013) |
|  | KM255003 | Saunders (2014) |  | HQ610379 | Saunders and Kucera (2010) |
|  | HQ610279 | Saunders and Kucera (2010) |  | KF195528 | Lawton et al. (2013) |
| *Ulva torta* | HQ610437 | Saunders and Kucera (2010) |  | HE600190 | Wolf et al. (2012) |
|  | MK992135 | Kang et al. (2019) | *Ulva stenophylla* | HQ610434 | Saunders and Kucera (2010) |
|  | MK992178 | Kang et al. (2019) | *Ulva prolifera* | EF595334 | Rinkel et al. (2012) |
| *Ulva procera* | MK992100 | Kang et al. (2019) | *Ulva linza* | HQ610368 | Saunders and Kucera (2010) |
|  | MK992088 | Kang et al. (2019) | *Ulva flexuosa* | HQ610296 | Saunders and Kucera (2010) |
|  | MK992078 | Kang et al. (2019) |  | JN029308 | Kirkendale et al. (2013) |
|  | MK992108 | Kang et al. (2019) | *Ulva* sp. | MK992193 | Kang et al. (2019) |
|  | MK992067 | Kang et al. (2019) |  | MK992144 | Kang et al. (2019) |
|  | HQ610393 | Saunders and Kucera (2010) | *Bliadingia sp.* | MK992087 | Kang et al. (2019) |
|  | MK992118 | Kang et al. (2019) |  | MK992131 | Kang et al. (2019) |
| *Ulva lobata* | HQ610375 | Saunders and Kucera (2010) |  | HQ610240 | Saunders and Kucera (2010) |

**Table S4** Results of partition by ABGD analyses for *tuf*A markers. P, Prior intraspecific divergence (P); IP, Initial partition; RP, Recursive partition; Ng, Number of groups of the full data set (Ng).

| Population | Jeju Island | | | | | | southern coasts | | | | | |
| --- | --- | --- | --- | --- | --- | --- | --- | --- | --- | --- | --- | --- |
| Locus | *tuf*A | | | | | | *tuf*A | | | | | |
| relative gap width (X) | X=1.0 | | X=1.5 | | X=2.0 | | X=1.0 | | X=1.5 | | X=2.0 | |
| Prior intraspecific divergence (P) | IP | *RP* | IP | *RP* | IP | *RP* | IP | *RP* | IP | *RP* | IP | *RP* |
| 0.0010 | 57 | - | 57 | - | 57 | - | 42 | - | 42 | - | 42 | - |
| 0.0017 | 23 | 25 | 20 | 21 | 10 | 19 | 16 | 19 | 16 | 19 | 16 | 19 |
| 0.0028 | 23 | 25 | 20 | 21 | 10 | 19 | 16 | 19 | 16 | 19 | 16 | 19 |
| 0.0046 | 20 | - | 20 | - | 10 | 13 | 16 | 17 | 16 | 17 | 16 | - |
| 0.0077 | 10 | 13 | 10 | 13 | 10 | 12 | 16 | - | 3 | - | 2 | 3 |
| 0.0129 | 10 | - | 10 | - | 10 | - | 13 | 14 | 3 | - | 2 | 3 |
| 0.0215 | 9 | - | 3 | 9 | 3 | 9 | 3 | - | 2 | 3 | 2 | 3 |
| 0.0359 | 9 | - | 3 | 9 | 3 | 9 | 3 | - | 2 | 3 | 2 | 3 |
| 0.0599 | 3 | 4 | 3 | 4 | 3 | 4 | 2 | 3 | 2 | 3 | 2 | 3 |
| 0.1000 | 1 | - | 1 | - | - | - | 2 | - | 2 | - | 2 | - |

**Table S5** Seasonal variation in *Ulva* community structure of Jeju Island.

| Species | 2019.11 | 2020.05 | 2020.07 | 2021.02 |
| --- | --- | --- | --- | --- |
| *U. australis* | 6.45 | 36.42 | 37.93 | 33.11 |
| *U. ohnoi* | 59.68 | 37.09 | 45.98 | 37.09 |
| *U. lactuca* | 28.23 | 0.00 | 2.87 | 0.00 |
| *U. californica* | 1.61 | 11.26 | 2.87 | 4.64 |
| *U. lacinulata* | 0.00 | 9.93 | 6.32 | 2.65 |
| *U. aragoënsis* | 0.81 | 1.32 | 1.72 | 3.97 |
| *U. arasakii* | 0.00 | 1.32 | 0.00 | 0.00 |
| *U. compressa* | 0.00 | 0.00 | 1.72 | 2.65 |
| *U. linza* | 1.61 | 1.32 | 0.00 | 12.58 |
| *U. prolifera* | 1.61 | 0.00 | 0.00 | 0.00 |
| *Ulva* sp. | 0.00 | 1.32 | 0.57 | 3.31 |

**Table S6** Seasonal variation in *Ulva* community structure of the southern coasts.

| Species | 2021.01 | 2021.04 | 2021.06 | 2021.10 |
| --- | --- | --- | --- | --- |
| *U. australis* | 46.15 | 32.17 | 45.05 | 15.52 |
| *U. ohnoi* | 1.28 | 3.48 | 1.10 | 43.10 |
| *U. lactuca* | 0.00 | 0.00 | 0.00 | 12.07 |
| *U. californica* | 5.13 | 2.61 | 5.49 | 10.34 |
| *U. lacinulata* | 5.13 | 13.04 | 4.40 | 6.90 |
| *U. aragoënsis* | 0.00 | 2.61 | 13.19 | 3.45 |
| *U. arasakii* | 3.85 | 0.87 | 0.00 | 0.00 |
| *U. compressa* | 0.00 | 6.09 | 0.00 | 0.00 |
| *U. linza* | 33.33 | 29.57 | 6.59 | 0.00 |
| *U. prolifera* | 5.13 | 6.96 | 0.00 | 0.00 |
| *Ulva* spp. | 0.00 | 2.61 | 24.18 | 8.62 |

**Supplementary Figures**

**Figure S1** Comparisons of water quality parameters across four groups: Group 1 (Jeju Island) and Groups 2, 3, and 4 (Wando, Yeosu, and Geoje, representing the southern coasts), based on data collected in February 2021 (MEIS^64^). Statistical analysis was performed using the Kruskal-Wallis test with Dunn's post hoc analysis and Bonferroni correction.


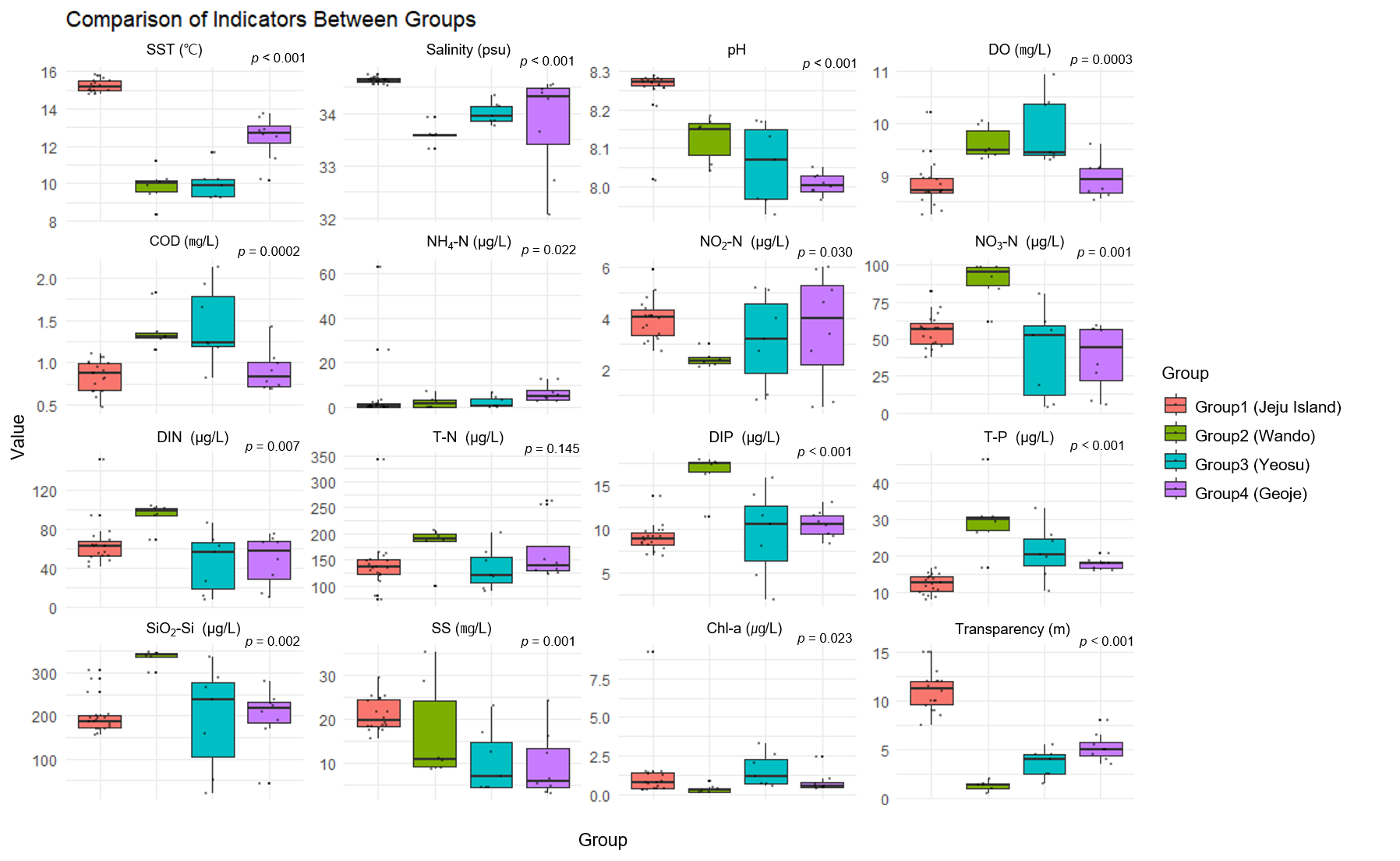


**Figure S2** Neighbor-joining (NJ) phylogeny based on 1,023 *tuf*A sequences (966 specimens from Jeju Island and the southern coasts combined, plus 55 previously determined haplotype sequences and two unidentified haplotypes) of *Ulva*, with two sequences of *Blidingia* species as the outgroup. Reference sequences were obtained from GenBank and used for phylogenetic analysis to identify *Ulva* species. Specimens grouped within particular clades are labeled as “Group” with the total number of specimens indicated [e.g., Group 1 (N=40)]. Bootstrap values are shown on the nodes for both maximum likelihood (ML) and neighbor-joining (NJ) methods.


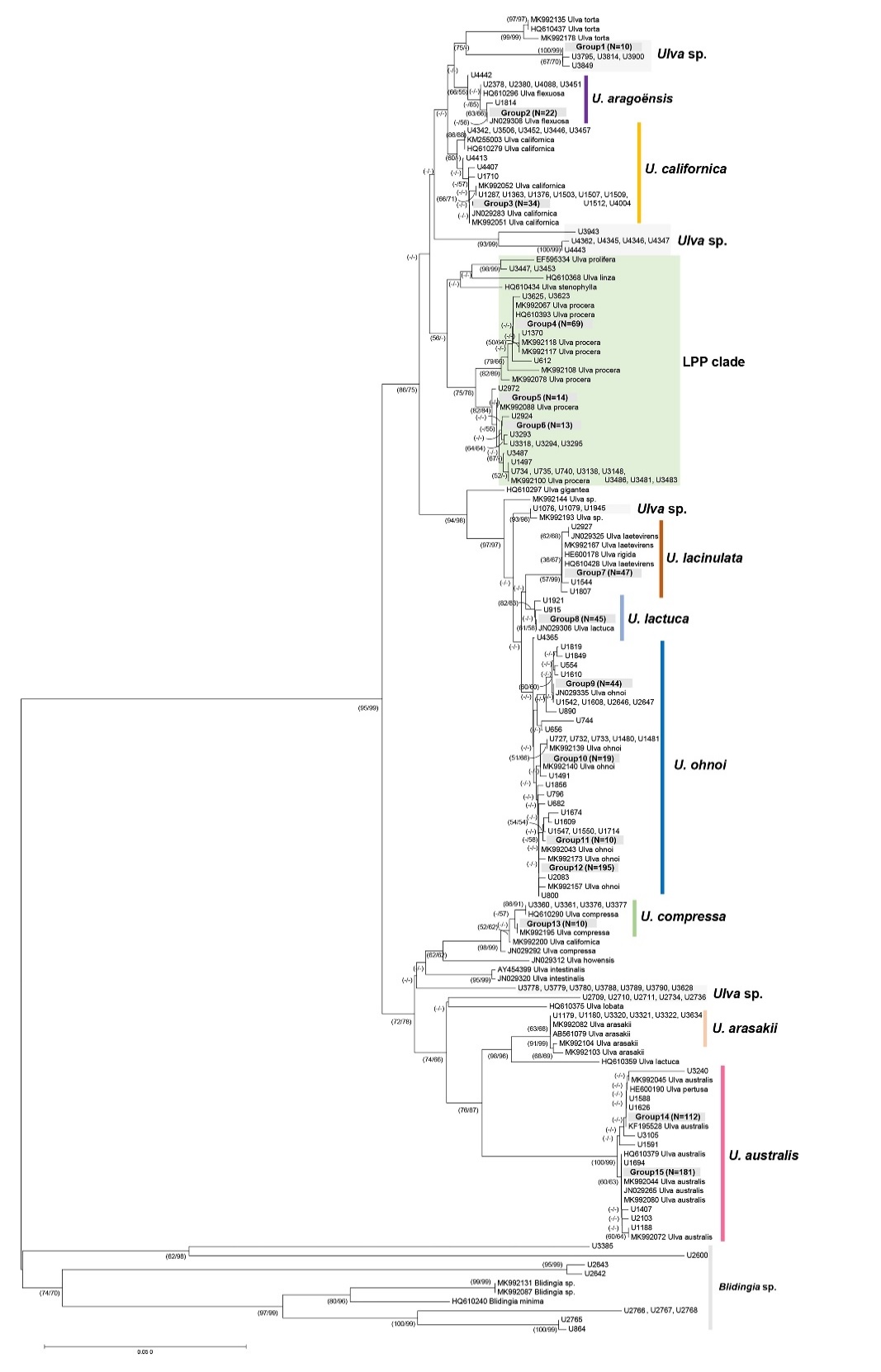


**Figure S3** Proportion of benthic and floating individuals of *U. ohnoi* (N=209) and *U. australis* (= *U. pertusa*) (N=129) on Jeju Island. Proportions were calculated from relative abundance counts. Results of Chi-square tests (χ² = 16.51, p < 0.001) and preference index values (PI: *U. ohnoi* = 0.48, *U. australis* = 0.04) are provided. All the analyses were conducted in R (version 4.5.0; R Core Team, 2025).


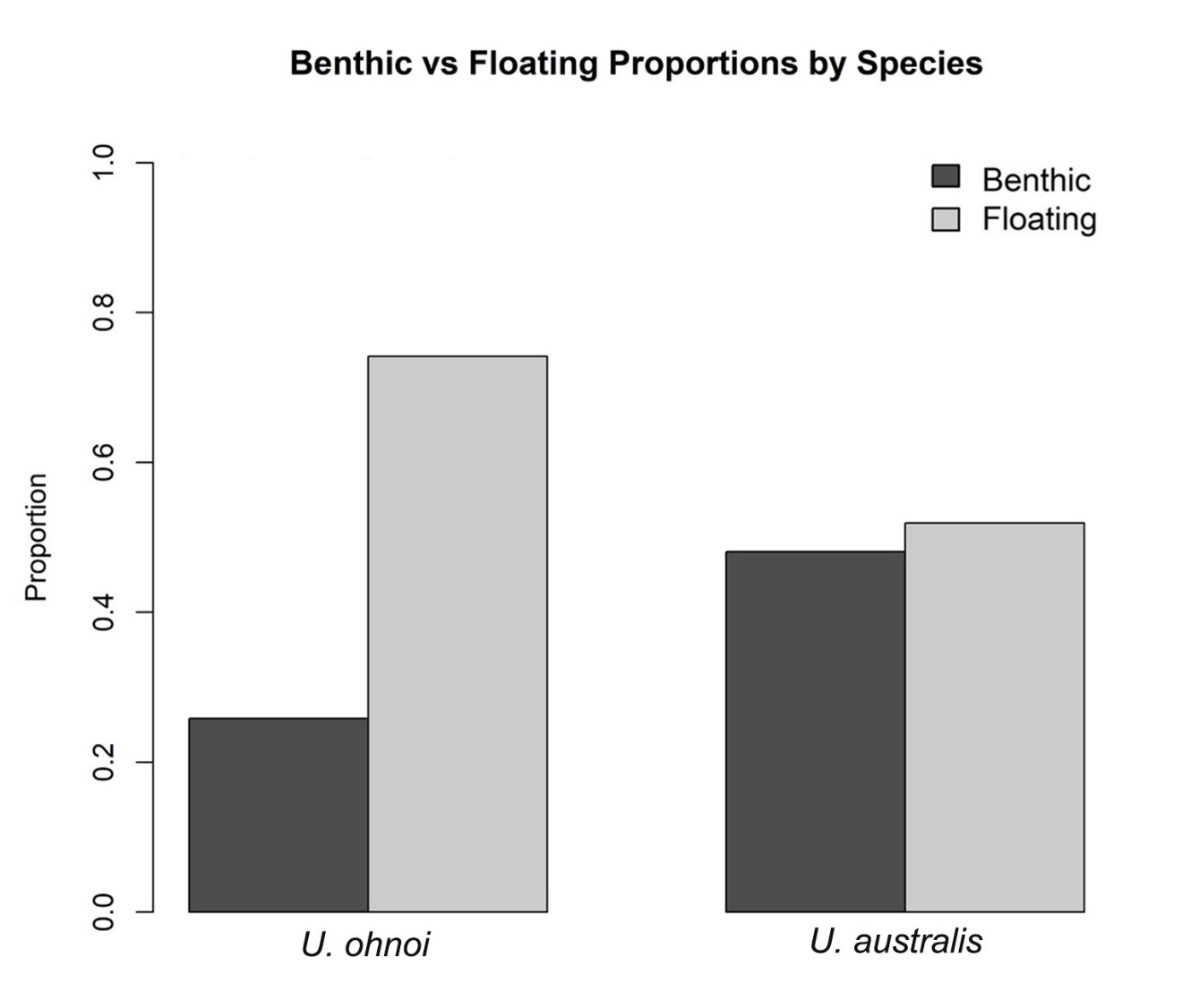

Supplement: Supplementary file 1 — Supplementary Material 1 [file 41598_2026_50151_MOESM1_ESM.docx]
